# Structure-Activity Relationships for the Anaesthetic and Analgaesic Properties of Aromatic Ring-Substituted Ketamine Esters

**DOI:** 10.3390/molecules25122950

**Published:** 2020-06-26

**Authors:** Ivaylo V. Dimitrov, Martyn G. Harvey, Logan J. Voss, James W. Sleigh, Michael J. Bickerdike, William A. Denny

**Affiliations:** 1 Auckland Cancer Society Research Centre, School of Medical Sciences, Auckland 1142, New Zealand; i.dimitrov@auckland.ac.nz; 2 Waikato Clinical School, University of Auckland, Private Bag 92019, Auckland 1142, New Zealand; Martyn.Harvey@waikatodhb.health.nz (M.G.H.); Logan.Voss@waikatodhb.health.nz (L.J.V.); Jamie.Sleigh@waikatodhb.health.nz (J.W.S.); 3 Kea Therapeutics Ltd, Level 20, Auckland 1010, New Zealand; Mike@keatx.com

**Keywords:** ketamine, esters, anaesthesia, short-acting, structure-activity relationship

## Abstract

A series of benzene ring substituted ketamine *N*-alkyl esters were prepared from the corresponding substituted norketamines. Few of the latter have been reported since they have not been generally accessible via known routes. We report a new general route to many of these norketamines via the Neber (oxime to α-aminoketone) rearrangement of readily available substituted 2-phenycyclohexanones. We explored the use of the substituents Cl, Me, OMe, CF_3_, and OCF_3_, with a wide range of lipophilic and electronic properties, at all available benzene ring positions. The 2- and 3-substituted compounds were generally more active than 4-substituted compounds. The most generally acceptable substituent was Cl, while the powerful electron-withdrawing substituents CF_3_ and OCF_3_ provided fewer effective analogues.

## 1. Introduction

**Figure 1:**
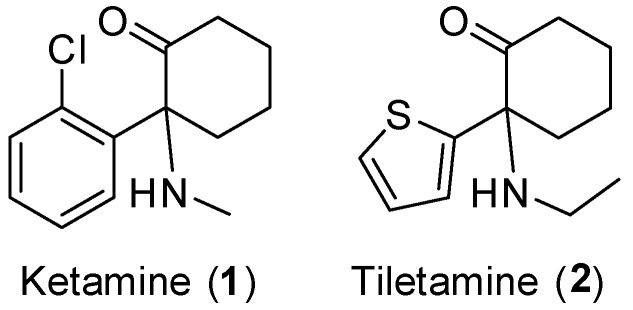
Ketamine, tiletamine.

Racemic ketamine (**1**, Figure 1) is an effective and widely used anaesthetic/analgaesic [1], and tiletamine (**2**) is a thiophene analogue widely used as an anaesthetic in veterinary medicine [2].

The sedative and analgaesic effects of **1** have commonly been attributed to its non-competitive antagonism of the calcium channel pore of the *N*-methyl-D-aspartate (NMDA) receptor [3,4], although this has recently been called into question [5]. Compared to opioid-type pain-relieving drugs, ketamine has the major advantages of no immediate respiratory depression or hyperalgesic effects, and an absence of longer-term effects such as increased tolerance [6]. The primary drawback of **1** is its substantial psychotogenic effects, which have recently been attributed to its blockade of GluN2C-containing NMDA receptors [7]. These detrimental properties are exacerbated by its relatively long elimination half-life, which means that patients can be exposed to prolonged hallucinogenic events as levels of drug slowly decline. To control these effects ketamine is frequently co-administered with respiratory depressant hypnotic drugs like midazolam or propofol, but these can markedly reduce its clinical safety [8]. In an alternative approach, we have recently shown, in a rat infusion model [9], that alkyl ester derivatives of ketamine (e.g. **5a**, **5b**) are effective short-term anaesthetics/analgaesics. They minimise psychotomimetic side effects during recovery by undergoing very rapid metabolism by tissue esterases to the corresponding, much more polar, and inactive acids [10]. Ester side chains (CH_2_)_2_CO_2_*^i^*Pr and (CH_2_)_4_CO_2_Me were particularly suitable [9,11].

We now extend these structure-activity studies to include analogues with Cl, Me, OMe, CF_3_ and OCF_3_ substituted at each available position on the benzene ring, together with the unsubstituted ring and 2-F variants (Table 1). Such substituents can potentially greatly affect drug binding to target proteins through the lipophilic, electronic, and steric changes that they have on the molecule. They collectively cover a wide range of lipophilic and electronic properties while keeping the steric effect broadly similar [12]. For further comparison we also included the corresponding esters (**20a**, **20b**) of the veterinary thiophene analogue tiletamine.

## 2. Chemistry and Biology 

### 2.1. Chemistry

**Scheme 1:**
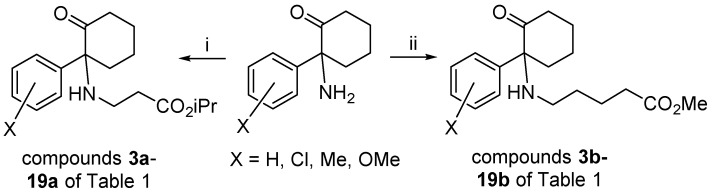
Synthesis of ketamine alkyl esters; Reagents and conditions: (i) Br(CH_2_)_2_CO_2_*^i^*Pr, K_2_CO_3_, KI, MeCN, reflux 12–48h; (ii) Br(CH_2_)_4_CO_2_Me, KI, MeCN, reflux 12–48h.

The synthesis of the compounds of Table 1 from the corresponding norketamines is straightforward as we have previously demonstrated [9]. (Scheme 1).

**Figure 2:**
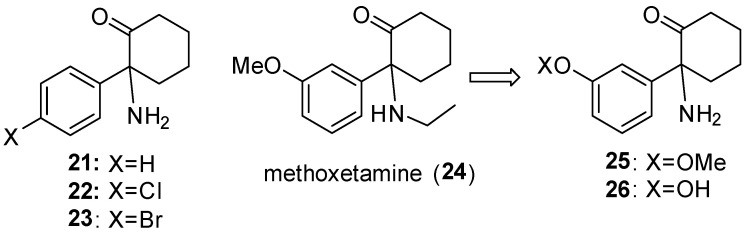
Known norketamine analogues.

However, few analogues of norketamine with substituents other than a 2-Cl in the aromatic ring have been reported; only the unsubstituted compound **21** [13] and the 4-Cl (**22**) and 4-Br (**23**) [14] analogues. The 3-OMe (**25**) and 3-OH (**26**) derivatives have also been characterised, but only as metabolites of methoxetamine (**24**) [15] (Figure 2).

**Scheme 2:**
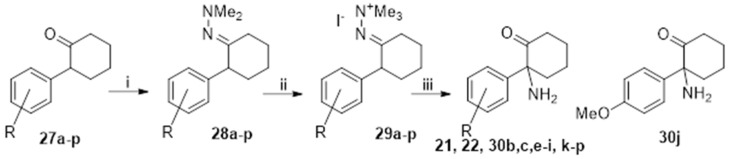
Synthesis of ring-substituted norketamines **21, 22, 30b,c, e–i, k–p.** R = : **a**, H; **b**, 2-F; **c**, 3-Cl; **d**, 4-Cl; **e**, 2-Me; **f**, 3-Me; **g**, 4-Me; **h**, 2-OMe; **i**, 3-OMe; **j**, 4-OMe; **k**, 2-CF_3_; **l**, 3-CF_3_, **m**, 4-CF_3_; **n**, 2-OCF_3_; **o**, 3-OCF_3_; **p**, 4-OCF_3_; Reagents and conditions: (i) NH_2_NMe_2_, EtOH, reflux 18 h; (ii) (a) MeI, MeCN, 40 °C for 2 h then 70 °C for 3 h; (iii) NaOEt, EtOH, reflux, 1 h.

We initially sought to prepare the required new substituted norketamines by the published method for norketamine itself [13] that we had used previously [9], but this was not successful, probably due to the lower nucleophilicity of ammonia compared with methylamine in that process. The use of more nucleophilic precursor reagents (*N*-methylhydrazine, 4-methoxybenzylamine) was also not successful. We therefore developed a new general route to many of these required norketamines, via the Neber (oxime to α-aminoketone) rearrangement [16] of substituted 2-phenycyclohexanones **27a–27p** via the hydrazines **28a–28p** and hydrazinium salts **29a**–**29p** to give the required norketamines **21, 22 and 30b,c**,**e–i, k–p** (Scheme 2).

The known 4-methoxynorketamine analogue **30j** could not be prepared by the Neber rearrangement, presumably because of the powerful electron-donating and/or inductive effects of this substituent para to the reaction centre, and was prepared instead by the method described by Sato et al [17].

**Scheme 3:**
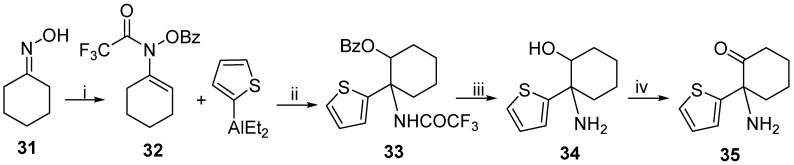
Synthesis of nortiletamine; Reagents and Conditions: (i) BzCl, pyridine, n-hexane-CH_2_Cl_2_ (10:1), 4 h r.t., then TFAA, CH_2_Cl_2_, r.t. 12 h, 89%; (ii) thiophene, *n*-BuLi, MeAlCl, THF, r.t. 30 min, then **32**, reflux, 3 h, 66%; (iii) NaOH (5% in MeOH), r.t. 12 h; (iv) Jones reagent, 84%.

In a further exploration of the nature of the aromatic ring in these esters, we also prepared the thiophene-based tiletamine ester analogues **20a** and **20b**. Tiletamine itself (**2**: Figure 1) is a well-known veterinary animal anaesthetic [2], considered to have a similar mechanism of action to ketamine. The required nortiletamine was prepared by the method of Sato et al. [17] (Scheme 3).

### 2.2. Biology

The compounds were evaluated for their ability to anaesthetise rats when administered by continuous intravenous infusion, as reported previously [9]. Compounds were administered to initially deliver 20 mg/kg/min (weight-adjusted flow) to achieve a pedal withdrawal reflex score (PWR = 1), then titrated to maintain loss of righting reflex (LORR) for 10 min. Three rats were used in each study, with each group of rats also acting as their own ketamine control. Data were collected on the total dose of drug (mg/kg), to achieve LORR and a PWR = 1, and on the time (in seconds, from cessation of the infusion) to recovery of righting reflex (RORR) (recovery from the hypnotic anaesthesia effect). Given the complexity of the experimental protocol, the total dose for LORR (Table 1) is very consistent, with ranges of only 1.5-fold within each group. The consistency of the post-sedation recovery times are expectedly lower, with ranges of about 2.5-fold. Average data for LORR and RORR are given in Table 1. During recovery the rats were monitored for multiple signs of behavioural dysfunction (see biology section for details) and the sum of these scores (from 0 to 4, with 0 being no effect and 4 being severe dysfunction) is given in Table 2. IC_50_ values for inhibition of the NMDA receptor were conducted by Eurofins PanLabs, Taiwan.

## 3. Results and Discussion

Ketamine has long been of interest for its multiple biological activities and its potential as a non-opioid anaesthetic. While a large number of side-chain analogues are known, relatively few with benzene ring substituents have been reported. The new route that we report here by the Neber rearrangement [16] of readily available substituted 2-phenycyclohexanones gives access to a wide range of benzene-substituted ketamines. We compare two sets of *N*-alkyl esters as short-acting anaesthetics, exploring Cl, Me, OMe, CF_3_, and OCF_3_ benzene ring substituents.

Table 1 gives structural and biological data for ketamine (**1**), two previously-reported [9,10] ester analogues (**5a**, **5b**), two series of esters of novel benzene ring-substituted analogues (**4a**–**19a**, **4b**–**19b**) and two similar esters (**20a**, **20b**) of the thiophene-based analogue tiletamine (**2**, Figure 1) [12].

It has often been stated, [3,4] and equally disputed [5], that the sedative analgaesic and psychotomimetic properties of ketamine are due to interaction with/inhibition of the NMDA receptor. The data acquired in this study for ketamine (**1**) and the ketamine esters (**3a–19a**, **3b–19b**) provide an opportunity to test these claims.

Of the 34 compounds studied (compound **17b** was omitted due to its seizure-inducing effect), 12 showed no sedative or analgaesic activity at doses up to 200 mg/kg. All but one of these compounds (**9b**) also showed no psychotomimetic properties, as judged by the behavioural dysfunction test. They also showed much weaker inhibition of the NMDA receptor than ketamine (IC_50_ 0.7 μM), with IC_50_s ranging from 57 to >1000 (average IC_50_ 350 μM).

In contrast, the 22 actively sedative compounds (including ketamine and the previously reported ester analogues **5a** and **5b**) had a wide range of IC_50_s for inhibition of NMDA (from 0.7 to >1000 μM) but did have a much lower average IC_50_ (167 μM). All but compounds **5a** and **17a** also showed analgaesic activity (most at potencies <60 mg/kg). This is broadly consistent with some relationships between these properties and NMDA inhibition. The majority of the active compounds were also much less psychotomimetic than ketamine (behavioural dysfunction score 3), but this may be at least in part due to the much faster recovery times for the esters (the main reason for this work). The shorter-chain ester analogue **20a** of tiletamine (**2**) was not active, but the longer-chain analogue **20b** was also an effective and relatively potent anaesthetics and analgaesics. However, **20b** generated severe dysfunction on awakening (score 4).

In terms of sedative activity structure-activity relationships for the benzene ring substituents, the active (CH_2_)_4_CO_2_Me series compounds were on average about 2.5-fold more potent than the corresponding (CH_2_)_2_CO_2_*^i^*Pr shorter-chain series, but the ring substituent effects were broadly similar across both series. The 2- and 3-substituted compounds were generally more active than 4-substituted compounds. The active anaesthetic compounds with the shorter (CH_2_)_2_CO_2_*^i^*Pr chain (**4a**–**19a**) included all of the 2-substituted examples except 2-Me (**6a**), making this overall the favoured position for substitution. The 3-Cl, 3-Me and 3-OMe compounds (**10a**–**12a**) and the 4-Cl and 4-OMe (**15a**, **16a**) analogues were also active anaesthetics. Overall, the most generally acceptable substituent was Cl, while the non-polar and powerful electron-withdrawing substituents CF_3_ and OCF_3_ were the least successful. All of the compounds generated very little dysfunction in the rats during recovery (averaged scores of mostly 0 or 1, of short duration), in contrast to ketamine (average score 3 for a prolonged period).

Overall, this study has helped to define the SAR for this series of ketamine esters and provide useful information towards selection of a clinical candidate.

### Conclusions

The above results show that the short chain aliphatic ester analogues of ketamine across the range of different benzene ring substituted compounds broadly retain the parent’s desirable anaesthetic and analgaesic properties, yet are sufficiently rapidly metabolised to minimise the drawbacks of ketamine in this capacity. The structure activity relationships for the esters were not straightforward, the results suggest the (CH_2_)_4_CO_2_Me series compounds were on average more active than the corresponding (CH_2_)_2_CO_2_*^i^*Pr shorter-chain series. The 2- and 3-substituted compounds were generally more active than 4-substituted compounds.

## 4. Experimental

### 4.1. Chemistry

All reagents and solvents were obtained from commercial suppliers and were used without further purification. Reactions requiring anhydrous conditions were performed under nitrogen atmospheres. Reactions were monitored by thin layer chromatography (TLC) on preloaded silica gel F254 plates (Merck, Darmstadt, Germany). with a UV indicator. Column chromatography was performed with Merck 230–400 mesh silica gel. ^1^H and ^13^C NMR spectra were obtained with a Bruker Avance 400 spectrometer (Bruker, Zuerich, Switzerland) at 400 MHz for ^1^H and 101 MHz for ^13^C spectra. Spectra were obtained in CDCl_3_ or (CD_3_)_2_SO. The chemical shifts are reported in parts per million (δ) downfield using tetramethylsilane (SiMe_4_) as internal standard. Spin multiplicities are given as s (singlet), d (doublet), dd (double doublet), br (broad), m (multiplet), and q (quartet). Coupling constants (J values) were measured in hertz (Hz). All LC/MS data were gathered by direct injection of methanolic solutions into a Surveyor MSQ mass spectrometer using an atmospheric pressure chemical ionisation (APCI) with a corona voltage of 50 V and a source temperature of 400 °C. High-resolution electrospray ionisation (HRESIMS) mass spectra were determined on a Bruker micrOTOFQ II mass spectrometer (Bruker, Switzerland). Final products were analysed by reverse-phase HPLC (Alltima C18 5 μm column, 150 mm × 3.2 mm; Alltech Associated, Inc., Deerfield, IL, USA) using an Agilent HP1100 equipped with a diode array detector. The mobile phase was 80% MeCN/20% H_2_O (*v*/*v*) in 45 mM HCO_2_NH_4_ at pH 3.5 and 0.5 mL/min. The purity was determined by monitoring at 272 nm and was ≥95% for final products unless otherwise stated. DCM refers to dichloromethane, DMF refers to *N,N*-dimethylformamide, EtOAc refers to ethyl acetate, EtOH refers to ethanol.

### 4.2. Synthesis of Ring-Substituted Norketamine Analogues. (Scheme 2)

**2-Amino-2-(4-chlorophenyl)cyclohexan-1-one (22).** A solution of 2-(4-chlorophenyl)cyclohexan-1-one (**27d**) (3.0 g, 14.4 mmol) and *unsym.*-dimethylhydrazine (3.46 g, 58.0 mmol), in EtOH (20 mL) was heated to 96 °C in a sealed tube for 12 h. The reaction mixture was cooled to room temperature, filtered and the solvent evaporated. The residue was purified by column chromatography on silica gel. Elution with EtOAc/hexanes (0–40%) gave 2-(2-(4-chlorophenyl)cyclohexylidene)-1,1-dimethylhydrazine (**28d**) (3.2 g, 90%) as a pale yellow oil. ^1^HNMR (CDCl_3_) δ 7.30–7.25 (m, 2H), 7.22–7.19 (m, 2H), 2.88–2.64 (dt, *J* = 13.96 Hz, 4.56 Hz, 1H), 2.47 (s, 6 H), 2.36–2.26 (m, 1H), 2.20–2.10 (m, 1 H), 2.00–1.92 (m, 1H), 1.82–1.72 (m 1H), 1.70–1.48 (m, 4H); MS *m/z* 251.20 (MH^+^).

A solution of **28d** (3.2 g, 12.8 mmol) and MeI (2.20 g, 15.4 mmol), in MeCN (20 mL) was heated in a sealed tube to 40 °C for 2 h, followed by heating to 70 °C for 3 h. The reaction mixture was cooled to room temperature, diluted with Et_2_O (60 mL) and left overnight in the fridge for the product to crystallise out. The solid was filtered and dried under high vacuum to yield the desired salt 2-(2-(4-chlorophenyl)cyclohexylidene)-1,1,1-trimethylhydrazinium iodide (**29d**) (4.99 g, 99%) as pale cream solid. ^1^HNMR (MeOD) δ 7.32–7.29 (m, 2H), 7.25–7.23 (m, 2H), 3.82–3.79 (q, *J* = 4.8 Hz, 1H), 3.46 (s, 9 H), 3.20–3.10 (m, 1H), 2.78–2.68 (m, 1H) 2.30–2.08 (m, 3H), 2.20–1.78 (m, 3H); MS *m/z* 251.20 ((MH-MeI)^+^).

Sodium (0.33g, 14.5 mmol), was washed with hexane, dried, cut into small pieces and placed in EtOH (40 mL) at r.t. The solution was stirred for approximately 20 min, until the sodium disappeared. The quaternary salt **29d** (5 g, 12 mmol) was added to the above solution and then it was refluxed for 1 h. The solution was cooled on ice and quenched with HCl (4 M, 40 mL). The ethanol was removed under reduced pressure, the residue was diluted with water (20 mL) and neutralised with NaOH (2 M) solution until pH 7. The aqueous layer was extracted with dichloromethane, MgSO_4_ dried and concentrated in vacuo. The residue was purified by column chromatography on silica gel eluting with EtOAc/hexanes (30–100%) to obtain 2-amino-2-(4-chlorophenyl)cyclohexan-1-one (**22**) (1.87 g, 70%) as a pale yellow oil. ^1^HNMR (CDCl_3_) δ 7.37–7.33 (m, 2H), 7.22–7.18 (m, 2H), 2.82–2.76 (m, 1H), 2.52–2.44 (m, 1H), 2.40–2.32 (m, 1H), 2.20–1.94 (m, 1H), 1.82–1.62 (m, 4H); MS *m/z* 224.20 (MH^+^).

Similarly were prepared:

**2-Amino-2-phenylcyclohexan-1-one (21).** Similar reaction of 2-phenylcyclohexan-1-one (**27a**) (2.43 g, 13.9 mmol) and *unsym.*-dimethylhydrazine gave 1,1-dimethyl-2-(2-phenylcyclohexylidene)hydrazine (**28a**) (2.62 g, 87%). ^1^HNMR (CDCl_3_) δ 7.30–7.29 (m, 3H), 7.21–7.19 (m, 2H), 3.01–2.95(dt, *J* = 13.84 Hz, 4.32 Hz, 1H), 2.50 (s, 6H), 2.33–2.32 (m, 1H), 2.07–1.91 (m, 2H), 1.81–1.70 (m, 2H), 1.69–1.60 (m, 1H), 1.59–1.52 (m, 2H); MS m/z 217.30 (MH^+^). Reaction of **28a** (2.62 g, 12.1 mmol) and methyl iodide as above gave 1,1,1-trimethyl-2-(2-phenylcyclohexylidene)hydrazin-1-ium iodide (**29a**) (3.0 g, 70%). ^1^HNMR (MeOD) δ 7.35–7.30 (m, 2H), 7.26–7.20 (m, 3H), 3.82–3.78 (dd, *J* = 9.49 Hz, 4.64 Hz, 1H), 3.47 (s, 9H), 3.05–2.89 (m, 1H), 2.82–2.76 (m, 1H), 2.36–2.30 (m, 1H), 2.18–2.15 (m, 1H), 2.08–2.03 (m, 1H), 1.97–1.79 (m, 3 H); MS m/z 217.2 ((MH-MeI)^+^). Reaction of **29a** (2.87 g, 8.0 mmol) with Na/EtOH as above then gave **21** (0.90 g, 60%). ^1^HNMR (CDCl_3_) δ 7.40–7.36 (m, 2H), 7.31–7.24 (m, 3H), 2.88–2.84 (m, 1H), 2.45–2.38 (m, 2H), 2.00–1.98 (m, 1H), 1.79–1.72 (m, 4H); MS m/z 190.20 (MH^+^).

**2-Amino-2-(2-fluorophenyl)cyclohexan-1-one (30b).** Similar reaction of 2-(2-fluorophenyl)cyclohexan-1-one (**27b**) (0.77 g, 4.0 mmol) and *unsym.*-dimethylhydrazine gave 2-(2-(2-fluorophenyl)cyclohexylidene)-1,1-dimethylhydrazine (**28b**) (0.72g, 82%) ^1^HNMR (CDCl_3_) δ 7.26–7.15 (m, 2H), 7.08–7.00 (td, *J* = 6.24 Hz, 1.24 Hz, 1 H), 6.98–6.95 (m, 1H), 3.86–3.74 (m, 1H), 3.10–3.04 (dt, *J* = 13.76 Hz, 4.40 Hz), 2.32 (s, 6 H), 2.28–2.19 (m, 2H), 2.08–1.98 (m, 2H), 1.90–1.78 (m, 2H), 1.68–1.56 (m, 2H); MS *m/z* 235.20 (MH^+^). Reaction of (**28b**) (0.66 g, 2.80 mmol) and methyl iodide as above gave 2-(2-(2-fluorophenyl)cyclohexylidene)-1,1,1-trimethylhydrazin-1-ium iodide (**29b**) (0.97 g, 94%) ^1^HNMR (MeOD) δ 7.31–7.24 (m, 2H), 7.15–7.11 (td, *J* = 7.56 Hz, 1.20 Hz, 1H), 7.07–7.02 (m, 1H), 4.01–3.97 (t, *J* = 8.52 Hz, 1H), 3.42 (s, 9H), 2.71–2.63 (m, 1H), 2.23–2.20 (m, 3H), 2.04–2.00 (m, 1H), 1.84–1.79 (m, 3H); MS *m/z* 235.30((MH-MeI)^+^). Reaction of **29b** (0.97 g, 2.60 mmol) with Na/EtOH as above then gave **30b** (0.37 g, 70%). ^1^HNMR (CDCl_3_) δ 7.53–7.48 (td, *J* = 7.80 Hz, 1.72 Hz, 1H), 7.33–7.28 (m, 1H), 7.22–7.18 (td, *J* = 7.68 Hz, 1.32 Hz, 1H), 7.07–7.02 (dd, *J* = 8.16 Hz, 1.24 Hz, 1H), 2.80–2.75 (m,1 H), 2.57–2.52 (m, 1H), 2.48–2.43 (m, 1H), 2.00–1.98 (m, 1H), 1.83–1.65 (m, 4H); MS *m/z* 208.20 (MH^+^).

**2-Amino-2-(3-chlorophenyl)cyclohexan-1-one (30c).** Similar reaction of 2-(3-chlorophenyl)cyclohexan-1-one (**27c**) (3.0 g, 14.4 mmol) and *unsym.*-dimethylhydrazine gave 2-(2-(3-chlorophenyl)cyclohexylidene)-1,1-dimethylhydrazine (**28c**) (3.2 g, 90%) as pale yellow oil. ^1^HNMR (CDCl_3_) δ 7.30–7.25 (m, 2H), 7.22–7.19 (m, 2H), 2.88–2.64 (dt, *J* = 13.96 Hz, 4.56 Hz, 1H), 2.47 (s, 6 H), 2.36–2.26 (m, 1H), 2.20–2.10 (m, 1 H), 2.00–1.92 (m, 1H), 1.82–1.72 (m 1H), 1.70–1.48 (m, 4H); MS *m/z* 251.20 (MH^+^). Reaction of **28c** (3.2 g, 12.8 mmol) and methyl iodide as above gave 2-(2-(3-chlorophenyl)cyclohexylidene)-1,1,1-trimethylhydrazinium iodide (**29c**) (4.99 g, 99%) as a solid. ^1^HNMR (MeOD) δ 7.29–7.27 (m, 2H), 7.25–7.24 (m, 1H), 7.19–7.17 (m, 1H), 3.84–3.79 (q, *J* = 5.12 Hz, 1H), 3.46 (s, 9H), 3.20–3.15 (m, 1H), 2.70–2.69 (m, 1 H), 2.22–2.12 (m, 3H), 1.98–1.96 (m, 1H), 1.84–1.82 (m, 2H); MS m/z 251.20 ((MH-MeI)^+^). Treatment of **29c** (3.8 g, 9.6 mmol) with Na/EtOH as above then gave **30c** 1.8 g, 84%) as an yellow oil. ^1^H NMR (CDCl_3_) δ 7.29–7.28 (m, 2 H), 2.27–7.26 (m, 1 H), 7.14–7.11 (dt, J = 7.36 Hz, 1.68 Hz, 1H), 2.82–2.78 (m, 1H), 2.52–2.48 (m, 1H), 2.40–2.36 (m, 1H), 2.04–2.00 (m, 1H), 1.80–1.72 (m, 4H); MS *m/z* 224.20 (MH)^+^.

**2-Amino-2-(*o*-tolyl)cyclohexan-1-one (30e).** Similar reaction of 2-(*o*-tolyl)cyclohexan-1-one **27e** (3.0 g, 16.0 mmol) and *unsym*.-dimethylhydrazine gave 1,1-dimethyl-2-(2-(*o*-tolyl)cyclohexylidene)hydrazine (**28e**) (3.65 mg, 99%). ^1^HNMR (CDCl_3_) δ 7.25–7.17 (m, 1H), 7.16–7.13 (m, 1H), 7.12–7.08 (m, 1H), 3.16–3.11 (dt, *J* = 10.4 Hz, 4.44 Hz, 1H), 2.34 (s, 6H), 2.26 (s, 3H), 2.12–2.02 (m, 2H), 2.00–1.82 (m, 3 H), 1.68–1.58 (m, 3H); MS *m/z* 231.30 (MH)^+^. Reaction of **28e** (3.65 g, 16.0 mmol) and methyl iodide as above gave 1,1,1-trimethyl-2-(2-(*o*-tolyl)cyclohexylidene)hydrazin-1-ium iodide (**29e**) (5.1 g, 86%). ^1^HNMR (MeOD) δ 7.20–7.18 (m, 1H), 7.16–7.08 (m, 3H), 3.95–3.91 (m, 1H), 3.39 (s, 9H), 2.74–2.66 (m, 1H), 2.26–2.21 (m, 3H), 2.24 (s, 3H), 2.10–1.98 (m, 1H), 1.92–1.78 (m, 2H); MS *m/z* 231.30 ((MH-MeI)^+^). Reaction of **29e** (5.10 g, 13.7 mmol) with Na/EtOH as above then gave **30e** (2.0 g, 72%). ^1^HNMR (CDCl_3_) δ 7.56–7.54 (dd, *J* = 8.76 Hz, 1.12 Hz, 1H), 7.27–7.18 (m, 3H), 2.92–2.88 (m, 1H), 2.52–2.36 (m, 1H), 2.17 (s, 3H), 2.04–1.93 (m, 2H), 1.76–1.72 (m, 4H); MS *m/z* 204.20 (MH)^+^.

**2-Amino-2-(*m*-tolyl)cyclohexan-1-one (30f).** Similar reaction of 2-(*m*-tolyl)cyclohexan-1-one (**27f**) (0.89 g, 4.70 mmol) and *unsym*.-dimethylhydrazine gave 1,1-dimethyl-2-(2-(*m*-tolyl)cyclohexylidene)hydrazine (**28f**) (0.9 g, 90%). ^1^HNMR (CDCl_3_) δ 7.23–7.16 (m, 1H), 7.12–7.08 (m, 1H), 7.04–6.98 (m, 2H), 3.02–2.96 (dt, *J* = 9.52 Hz, 4.21 Hz, 1H), 2.52 (s, 6H), 2.48 (s, 3H), 2.08–1.88 (m, 2H), 1.86–1.50 (m, 6H); *m/z* 231.3 (MH+). Reaction of **28f** (0.9 g, 3.90 mmol) and methyl iodide as above gave 1,1,1-trimethyl-2-(2-(*m*-tolyl)cyclohexylidene)hydrazin-1-ium iodide (**29f**) (1 g, 70%). ^1^HNMR (MeOD) δ 7.21–7.17 (t, *J* = 7.64 Hz, 1H), 7.07–7.04 (t, *J* = 6.92 Hz, 3H), 3.78–3.74 (dd, *J* = 9.24 Hz, 4.6 Hz, 1H), 3.48 (s, 9H), 3.02–2.96 (m, 1H), 2.86–2.74 (m, 1H), 2.40–2.30 (m, 1H), 2.36 (s, 3H), 2.18–2.00 (m, 2H), 2.00–1.76 (m, 3H); MS *m/z* 231.20 ((MH-MeI)^+^). Reaction of **29f** (0.83 g, 2.23 mmol) with Na/EtOH as above then gave **30f** (0.3 g, 67%). ^1^HNMR (CDCl_3_) δ 7.28–7.24 (t, *J* = 8.3 Hz, 1H), 7.11–7.09 (m, 1H), 7.07–7.05 (m, 2H), 2.90–2.82 (m, 1H), 2.48–2.40 (m, 2H), 2.34 (s, 3H), 2.04–1.98 (m, 1H), 1.82–1.62 (m, 4H); MS *m/z* 204.2 (MH)^+^.

**2-Amino-2-(*p*-tolyl)cyclohexan-1-one (30g).** Similar reaction of 2-(*p*-tolyl)cyclohexan-1-one (**27g**) (3 g, 16.0 mmol) and *unsym*.-dimethylhydrazine gave 1,1-dimethyl-2-(2-(*p*-tolyl)cyclohexylidene)hydrazine (**28g**) (3.07 g, 83%). ^1^H NMR (CDCl_3_) δ 7.18–7.16 (m, 2H), 7.14–7.10 (m, 2H), 2.99–2.94 (dt, *J =* 9.8 Hz, 4.28 Hz, 1H), 2.50 (s, 6H), 2.30 (s, 3H), 2.08–1.89 (m, 2H), 1.82–1.60 (m, 4H), 1.60–1.49 (m, 2 H); MS *m/z* 231.20 (MH)^+^. Reaction of **28g** (3.07 g, 13.3 mmol) and methyl iodide as above gave 2-(2-(*p*-tolyl)cyclohexylidene)-1,1,1-trimethylhydrazinium iodide (**29g**) (3.72 g, 75%). ^1^H NMR (MeOD) δ 7.24–7.16 (m, 1H), 7.15–7.13 (m, 3H), 3.77–3.74 (m, 1H), 3.48 (s, 9H), 3.02–2.94 (m, 1H), 2.84–2.74 (m, 1H), 2.40–2.32 (m, 1H), 2.30 (s, 3H), 2.19–2.08 (m, 1H), 2.06–1.98 (m, 1H), 1.94–1.84 (m, 2H), 1.86–1.76 (m, 1H); MS *m/z* 231.20 ((MH-MeI)^+^). Reaction of **29g** (3.72 g, 10.0 mmol) with Na/EtOH as above then gave **30g** (1 g, 50%). ^1^HNMR (CDCl_3_) δ 7.20–7.18 (m, 2 H), 7.14–7.12 (m, 2H), 2.84 (br s, 1H), 2.35–2.30 (m, 2H), 2.34 (s, 3H), 2.20–2.00 (m, 1H), 1.80–1.70 (m, 4H); MS *m/z* 204.20 (MH)^+^.

**2-Amino-2-(2-methoxyphenyl)cyclohexan-1-one (30h)**. Similar reaction of 2-(2-methoxyphenyl)cyclohexan-1-one (**27h**) (3.0 g, 14.7mmol) and *unsym.*-dimethylhydrazine gave 2-(2-(2-methoxyphenyl)cyclohexylidene)-1,1-dimethylhydrazine (**28h**) (3.0 g, 83%). ^1^HNMR (CDCl_3_) δ 7.19–7.14 (m, 2H), 6.92–6.82 (m, 2H), 3.77 (s, 3H), 3.00–2.94 (m, 1H), 2.58–2.38 (m, 1H), 2.38 (s, 4H), 2.22–2.12 (m, 1H), 2.02–1.98 (m, 2H), 1.82–1.70 (m, 2H), 1.70–1.58 (m, 2H); MS *m/z* 247.20 (MH^+^). Reaction of **28h** (3.0 g, 12.2 mmol) and methyl iodide as above gave 2-(2-(2-methoxyphenyl)cyclohexylidene)-1,1,1-trimethylhydrazin-1-ium iodide (**29h**) (4.31 g, 91%). ^1^HNMR (MeOD) δ 7.24–7.17 (m, 2H), 6.94–6.88 (m, 2H), 4.01–3.97 (m, 1H), 3.82 (s, 3H), 3.38 (s, 9H), 2.70–2.62 (m, 2H), 2.22–2.14 (m, 2H), 2.08–2.00 (m, 2H), 1.90–1.74 (m, 2H); MS *m/z* 247.20 ((MH-MeI)^+^). Reaction of **29h** (1.00 g, 2.60 mmol) with Na/EtOH as above then gave **30h** (0.30 g, 53%). ^1^HNMR (CDCl_3_) δ 7.54–7.52 (dd, *J* = 7.76 Hz, 1.6 Hz, 1H), 7.32–7.27 (td, *J* = 7.48 Hz, 1.6 Hz, 1H), 7.06–7.01 (td, *J* = 7.6 Hz, 1.16 Hz, 1H), 6.90–6.87 (dd, *J* = 8.29 Hz, 0.92 Hz, 1H), 3.73 (s, 3H), 2.40–2.31 (m, 2H), 1.99–1.92 (m, 2H), 1.76–1.58 (m, 4H); MS *m/z* 220.20 (MH^+^).

**2-Amino-2-(3-methoxyphenyl)cyclohexan-1-one (30i).** Similar reaction of 2-(3-methoxyphenyl)cyclohexan-1-one (**27i**) (1.0 g, 4.90 mmol) and *unsym.*-dimethylhydrazine gave 2-(2-(3-methoxyphenyl)cyclohexylidene)-1,1-dimethylhydrazine (**28i**) (1.2 g, 100%). ^1^HNMR (CDCl_3_) δ 7.26–7.22 (m, 1H), 6.90–6.81 (m, 1H), 6.78–6.68 (m, 2H), 3.76 (s, 3H), 3.04–2.98 (dt, *J =* 13.81 Hz, 4.17 Hz, 1 H), 2.49 (s, 6H), 2.06–1.88 (m, 2H), 1.82- 1.70 (m, 2H), 1.70–1.62 (m, 2H), 1.60–1.50 (m, 2 H); MS *m/z* 247.20 (MH^+^). Reaction of **28i** (1.2 g, 4.88 mmol) and methyl iodide as above gave 2-(2-(3-methoxyphenyl)cyclohexylidene)-1,1-dimethylhydrazine (**29i**) (1.20 g, 63%). ^1^HNMR (MeOD) δ 7.26–7.21 (td, *J =* 9.12 Hz, 1.52 Hz, 1 H), 6.85–6.79 (m, 3H), 3.78 (s, 3H), 3.49 (s, 9H), 3.04–2.98 (m, 1H), 2.83–2.77 (m, 1H), 2.70–2.58 (m, 1H), 2.40–2.32 (m, 1H), 2.20–2.10 (m, 1H), 2.10–2.00 (m, 1H), 2.00–1.80 (m, 3H); MS *m/z* 247.20 ((MH-MeI)^+^). Reaction of **29i** (0.67 g, 1.72 mmol) with Na/EtOH as above then gave **30i** (0.20 g, 54%).^1^H NMR (CDCl_3_) ^1^HNMR (CDCl_3_) δ 7.31–7.26 (m, 1H), 6.84–6.82 (m, 3H), 3.79 (s, 3H), 2.83–2.81 (m, 1H), 2.44–2.40 (m, 2H), 2.04–1.95 (m, 2H), 1.76–1.71 (m, 3H); MS *m/z* 220.20 (MH^+^).

**2-Amino-2-(2-(trifluoromethyl)phenyl)cyclohexan-1-one (30k).** Similar reaction of 2-(2-(trifluoromethyl)phenyl)cyclohexan-1-one (**27k**) (1.28 g, 5.30 mmol) and *unsym.*-dimethylhydrazine gave 1,1-dimethyl-2-(2-(2-(trifluoromethyl)phenyl)cyclohexylidene)hydrazine (**28k**) (1.20, 80%) ^1^HNMR (CDCl_3_) δ 7.60–7.58 (d, *J* = 7.92 Hz, 1H), 7.46–7.42 (m, 2H), 7.29–7.27 (m, 1H), 3.82–3.78 (dd, *J* = 12.25 Hz, 4.36 Hz, 1H), 3.44–3.39 (m, 1H), 2.24 (s, 6H), 2.10–1.80 (m, 5H), 1.72–1.48 (m, 3H); MS *m/z* 285.20 (MH^+^). Reaction of **28k** (1.00 g, 3.50 mmol) and methyl iodide as above gave 1,1,1-trimethyl-2-(2-(2-(trifluoromethyl)phenyl)cyclohexylidene)hydrazin-1-ium iodide (**29k**) (1.44 g, 96%) ^1^HNMR (MeOD) δ 7.67–7.65 (d, *J* = 7.88 Hz, 1H), 7.62–7.52 (m, 2H), 7.44–7.40 (m, 1H), 4.09–4.05 (dd, *J* = 7.88 Hz, 4.36 Hz, 1H), 3.38 (s, 9H), 2.68–2.54 (m, 1H), 2.38–2.22 (m, 2H), 2.10–1.96 (m, 2H), 1.90–1.82 (m, 2H), 1.76–1.62 (m, 1H); MS *m/z* 285.20((MH-MeI)^+^). Reaction of **29k** (1.44 g, 3.40 mmol) with Na/EtOH as above then gave **30k** (0.62 g, 72%). ^1^HNMR (CDCl_3_) δ 7.98–7.96 (d, *J* = 8.05 Hz, 1H), 7.71–7.68 (dd, *J* = 7.88 Hz, 1.2 Hz, 1H), 7.61–7.57 (td, *J* = 7.52 Hz, 0.68 Hz, 1H), 7.52–7.42 (t, *J* = 7.64 Hz, 1H), 2.76–2.68 (m, 1H), 2.57–2.42 (m, 1H), 1.98–1.92 (m, 3H), 1.87–1.77 (m, 5H); MS *m/z* 258.20 (MH^+^).

**2-Amino-2-(3-(trifluoromethyl)phenyl)cyclohexan-1-one (30l).** Similar reaction of 2-(3-(trifluoromethyl)phenyl)cyclohexan-1-one (**27l**) (1.28 g, 5.30 mmol) and *unsym.*-dimethylhydrazine gave 1,1-dimethyl-2-(2-(3-(trifluoromethyl)phenyl)cyclohexylidene)hydrazine (**28l**) (1.2 g, 71%) ^1^HNMR (CDCl_3_) δ 7.54–7.50 (m,1H), 7.49–7.37 (m, 3H), 3.70–3.67 (t, *J* = 5.2Hz, 1H), 2.47 (s, 6H), 2.33–2.20 (m, 2H), 2.08–1.98 (m, 1H), 1.94–1.66 (m, 3H), 1.56–1.44 (m, 2H); MS *m/z* 285.20 (MH^+^). Reaction of **28l** (1.07 g, 2.80 mmol) and methyl iodide as above gave 1,1,1-trimethyl-2-(2-(3-(trifluoromethyl)phenyl)cyclohexylidene)hydrazin-1-ium iodide (**29l**) (1.40 g, 86%) ^1^HNMR (MeOD) δ 7.55–7.52 (m, 4H), 3.94–3.90 (m, 1H), 3.44 (s, 9H), 2.73–2.69 (m, 1H), 2.53–2.50 (m, 1H), 2.24–2.18 (m, 3H), 2.03–1.98 (m, 1H), 1.88–1.82 (m, 2H); MS *m/z* 285.20((MH-MeI)^+^). Reaction of **29l** (1.40 g, 3.30 mmol) with Na/EtOH as above then gave **30l** (0.52 g, 62%). ^1^HNMR (CDCl_3_) δ 7.58–7.55 (m, 1H), 7.52–7.49 (t, *J* = 7.72 Hz, 2H), 7.45–7.43 (m, 1H), 2.88–2.82 (m, 1H), 2.58–2.50 (m, 1H), 2.38–2.30 (m, 1H), 2.06–1.98 (m, 1H), 1.92–1.80 (m, 3H), 1.80–1.70 (m, 1H); MS *m/z* 258.20 (MH^+^).

**2-Amino-2-(4-(trifluoromethyl)phenyl)cyclohexan-1-one (30m).** Similar reaction of 2-(4-(trifluoromethyl)phenyl)cyclohexan-1-one (**27m**) (1.28 g, 5.30 mmol) and *unsym.*-dimethylhydrazine gave 1,1-dimethyl-2-(2-(4-(trifluoromethyl)phenyl)cyclohexylidene)hydrazine (**28m**) (1.0 g, 76%) ^1^HNMR (CDCl_3_) δ 7.58–7.54 (m, 2H), 7.40–7.37 (d, *J* = 8.56 Hz, 2H), 3.70–3.67 (t, *J* = 5.16 Hz, 1H), 2.86–2.78 (m, 1H), 2.48 (s, 6H), 2.38–2.28 (m, 1H), 2.28–2.18 (m, 1H), 2.20–1.96 (m, 1H), 1.82–1.60 (m, 4H); MS *m/z* 285.20 (MH^+^). Reaction of **28m** (1.00 g, 3.50 mmol) and methyl iodide as above gave 1,1,1-trimethyl-2-(2-(4-(trifluoromethyl)phenyl)cyclohexylidene)hydrazin-1-ium iodide (**29m**) (1.22, 82%) ^1^HNMR (MeOD) δ 7.65–7.59 (d, *J* = 13.36 Hz, 2H), 7.46–7.44 (d, *J* = 8.44 Hz, 2H), 4.84 (s, 9H), 3.92–3.88 (q, *J* = 4.88 Hz, 1H), 2.74–2.69 (m, 1H), 2.53–2.49 (m, 1H), 2.22–1.97 (m, 3H), 1.93–1.88 (m, 1H), 1.88–1.84 (m, 2H); MS *m/z* 285.20((MH-MeI)^+^). Reaction of **29m** (1.22 g, 2.80 mmol) with Na/EtOH as above then gave **30m** (0.48 g, 66%). ^1^HNMR (CDCl_3_) δ 7.65–7.63 (d, *J* = 8.25 Hz, 2H), 7.41–7.39 (d, *J* = 8.20 Hz, 2H), 2.88–2.80 (m, 1H), 2.58–2.50 (m, 1H), 2.40–2.32 (m, 1H), 2.04–1.98 (m, 1H), 1.88–1.72 (m, 4H); MS *m/z* 258.20 (MH^+^).

**2-Amino-2-(2-(trifluoromethoxy)phenyl)cyclohexan-1-one (30n).** Similar reaction 2-(2-(trifluoromethoxy)phenyl)cyclohexan-1-one (**27n**) (1.11 g, 5.30 mmol) and *unsym.*-dimethylhydrazine gave 2-(2,2-dimethylhydrazono)-1-(2-(trifluoromethoxy)phenyl)cyclohexan-1-amine (**28n**) (1.0 g, 78%) ^1^HNMR (CDCl_3_) δ 7.32–7.29 (m, 1H), 7.26–7.18 (m, 3H), 3.79–3.76 (dd, *J* = 12.40 Hz, 4.16 Hz, 1H), 2.27 (s, 6H), 2.04–1.82 (m, 5H), 1.72–1.52 (m, 3H); MS *m/z* 301.20 (MH^+^). Reaction of **28n** (1.00 g, 3.30 mmol) and methyl iodide as above gave 2-(2-amino-2-(2-(trifluoromethoxy)phenyl)cyclohexylidene)-1,1,1-trimethylhydrazin-1-ium iodide (**29n**) (1.35g, 92%) ^1^HNMR (MeOD) δ 7.44–7.42 (m, 1H), 7.36–7.32 (m, 2H), 7.28–7.26 (m, 1H), 4.07–4.03 (m, 1H), 3.40 (s, 9H), 2.69–2.66 (m, 1H), 2.26–2.19 (m, 3H), 2.06–2.03 (m, 2H), 1.84–1.79 (m, 2H); MS *m/z* 301.20((MH-MeI)^+^). Reaction of **29n** (1.35 g, 3.05 mmol) with Na/EtOH as above then gave **30n** (0.42 g, 52%). ^1^HNMR (CDCl_3_) δ 7.69–7.67 (m, 1H), 7.39–7.34 (m, 2H), 7.29–7.26 (m, 1H), 2.77–2.71 (m, 1H), 2.58–2.52 (m, 1H), 2.45–2.38 (m, 1H), 1.82–1.77 (m, 1H), 1.82–1.62 (m, 4H); MS *m/z* 274.20 (MH^+^).

**2-Amino-2-(3-(trifluoromethoxy)phenyl)cyclohexan-1-one (30o).** Similar reaction of 2-(3-(trifluoromethoxy)phenyl)cyclohexan-1-one (**27o)** (1.62 g, 5.40 mmol) and *unsym.*-dimethylhydrazine gave 2-(2,2-dimethylhydrazono)-1-(3-(trifluoromethoxy)phenyl)cyclohexan-1-amine (**28o**) (2.0 g, 84%) ^1^HNMR (CDCl_3_) δ 7.37–7.31 (m, 1H), 7.22–7.18 (m, 1H), 7.14–7.11 (m, 1H), 7.08–7.04 (m, 1H), 3.68–3.64 (t, *J* = 5.0 Hz, 1H), 2.48 (s, 6H), 2.34–2.28 (m, 1H), 2.20–2.10 (m, 1H), 2.06–1.96 (m, 1H), 1.94–1.88 (m, 1H), 1.80–1.50 (m, 4H); MS *m/z* 301.20 (MH^+^). Reaction of **28o** (1.62 g, 5.40 mmol) and methyl iodide as above gave 2-(2-amino-2-(3-(trifluoromethoxy)phenyl)cyclohexylidene)-1,1,1-trimethylhydrazin-1-ium iodide (**29o**) (2.00 g, 84%) ^1^HNMR (MeOD) δ 7.40–7.38 (t, *J* = 7.97 Hz, 1H), 7.30–7.26 (m, 1H), 7.22–7.18 (m, 1H), 7.17–7.11 (m, 1H), 3.89–3.85 (t, *J* = 7.73 Hz, 1H), 3.44 (s, 9H)HH, 2.52–2.44 (m, 1H), 2.24–2.14 (m, 2H), 2.04–1.96 (m, 2H), 1.92–1.78 (m, 2H), 1.70–1.60 (m, 1H); MS *m/z* 301.20((MH-MeI)^+^). Reaction of **29o** (2.00 g, 4.50 mmol) with Na/EtOH as above then gave **30o** (0.60 g, 50%). ^1^HNMR (CDCl_3_) δ 7.43–7.41 (td, *J* = 7.93 Hz, 0.96 Hz, 1H), 7.18–7.16 (m, 3H), 2.82–2.76 (m, 1H), 2.54–2.48 (m, 1H), 2.40–2.32 (m, 1H), 2.04–1.98 (m, 1H), 1.90–1.70 (m, 4H); MS *m/z* 274.20 (MH^+^).

**2-Amino-2-(4-(trifluoromethoxy)phenyl)cyclohexan-1-one (30p).** Similar reaction of 2-(4-(trifluoromethoxy)phenyl)cyclohexan-1-one (**27p**) (2.24 g, 8.68 mmol) and *unsym.*-dimethylhydrazine gave 2-(2,2-dimethylhydrazono)-1-(4-(trifluoromethoxy)phenyl)cyclohexan-1-amine (**28p**) (2.0 g, 76%) ^1^HNMR (CDCl_3_) δ 7.30–7.27 (m, 2H), 7.17–7.13 (m, 2H), 3.67–3.64 (t, *J* = 7.13 Hz, 1H), 2.89–2.58 (m, 1H), 2.45 (s, 6H), 2.32–2.14 (m, 2H), 2.02–1.94 (m, 1H) 1.80–1.58 (m, 4H); MS *m/z* 301.20 (MH^+^). Reaction of **28p** (1.82 g, 6.10 mmol) and methyl iodide as above gave 2-(2-amino-2-(4-(trifluoromethoxy)phenyl)cyclohexylidene)-1,1,1-trimethylhydrazin-1-ium iodide (**29p**) (2.62 g, 98%) ^1^HNMR (MeOD) δ 7.36–7.34 (m, 2H), 7.22–7.20 (d, *J* = 7.92 Hz, 2H), 3.89–3.85 (m, 1H), 3.46 (s, 9H), 2.78–2.70 (m, 1H), 2.58–2.49 (m, 1H), 2.30–2.10 (m, 3H), 2.00–1.80 (m, 3H); MS *m/z* 301.20((MH-MeI)^+^). Reaction of **29p** (2.62 g, 5.90 mmol) with Na/EtOH as above then gave **30p** (0.80 g, 50%). ^1^HNMR (CDCl_3_) δ 7.32–7.29 (m, 2H), 7.23–7.21 (m, 2H), 2.84–2.78 (m, 1H), 2.52–2.49 (m, 1H), 2.40–2.34 (m, 1H), 2.06–1.98 (m, 1H), 1.88–1.62 (m, 4H); MS *m/z* 274.20 (MH^+^).

### 4.3. Synthesis of Nortiletamine (35) (Scheme 3).

***N*-(Benzoyloxy)-*N*-(cyclohex-1-en-1-yl)-2,2,2-trifluoroacetamide (32)**. To a solution of cyclohexanone oxime (**31**) (5.5 g, 48.6 mmol) in *n*-hexane:DCM (160 mL, 10:1) was added pyridine (3.84 g, 48.6 mmol) and benzoyl chloride (6.8 g, 48.6 mmol) dropwise at room temperature. The mixture was stirred at room temperature for 4 h and then diluted with water (100 mL). The organic layer was washed with water (3 × 70 mL), dried (MgSO_4_) and concentrated in vacuo to afford the corresponding *O*-benzoyloxime ether (10.17 g, 96%), which was used in the subsequent reaction without purification. A solution of this in DCM (70 mL) was treated with TFAA (35 mL) dropwise at 0 °C. The mixture was stirred at room temperature for 12 h and then concentrated in vacuo. The residue was purified by flash chromatography (hexane-EtOAc, 50:1) to give enamide **32** (13 g, 89%). ^1^HNMR (CDCl_3_) δ 8.12–8.08 (d, *J* = 7.5 Hz, 2H), 7.66–7.59 (m, 1H), 7.51–7.44 (m, 2H), 6.36–6.20 (br m, 1H), 2.37–2.21 (m, 4H), 1.79–1.71 (m, 2H), 1.66–1.58 (m, 2H); MS *m/z* 314.10 (MH^+^).

**2-(Thiophen-2-yl)-2-(2,2,2-trifluoroacetamido)cyclohexyl benzoate (33)**. A solution of thiophene (2.67 g, 31.9 mmol) in THF (10 mL), was treated with *n*-BuLi (2M in cyclohexane, 15.95 mL, 31.9 mmol) dropwise at –78 °C. The mixture was stirred at 0 °C for 30 min and then Et_2_AlCl (1M in hexane, 31.9 mL, 31.9 mmol) was added. The mixture was then stirred for additional 30 min at room temperature. The resulting diethyl(thiophene)aluminium reagent was used directly in the subsequent reaction. A solution of enamide **32** (5 g, 15.9 mmol) in THF (60 mL) was added dropwise to the above-generated aluminium reagent at room temperature. The reaction was stirred under reflux for 3 h and quenched with 1.3 M aq. Rochelle’s salt (150 mL). The aqueous layer was DCM extracted (3x 100 mL) and the combined organic layers were washed with water (100mL). The residue was purified by flash chromatography (hexane-EtOAc, 5:1) to give **33** (4.14 g, 66%). ^1^HNMR (CDCl_3_) δ 7.95–7.93 (dm, *J* = 8.5 Hz, 2H), 7.61–7.57 (tm, *J* = 7.5 Hz, 1H), 7.48–7.44 (tm, *J* = 7.0 Hz, 2H), 7.16–7.14 (dd, *J =* 5.0 Hz, 1.5 Hz, 1H), 7.00–6.98 (dd, *J* = 3.5 Hz, 1.5 Hz, 2H), 6.89–6.87 (dd, *J* = 5.0 Hz, 3.5 Hz, 1H), 5.29–5.26 (dd, *J* = 10.5 Hz, 4.0 Hz, 1H), 3.32–3.28 (dm, *J* = 14.5 Hz, 1H), 2.16–2.04 (m, 2H), 1.89–1.67 (m, 3H), 1.62–1.39 (m, 2H); MS *m/z* 396.1 (M-H^+^).

**2-Amino-2-(thiophen-2-yl)cyclohexan-1-ol (34).** The *O*-benzoyl ester **33** (4.14 g, 10.4 mmol) was stirred with 5% NaOH in MeOH (200 mL) at room temperature for 16 h. The solvent was then removed in vacuo, the residue was dissolved in DCM (100 mL) and washed with water (3 × 200 mL). The residue was purified with flash chromatography eluting with EtOAc (100%) to yield amino alcohol **34** (1.8 g, 88%). ^1^HNMR (CDCl_3_) δ 7.21–7.19 (dm, *J* = 5.0 Hz, 1H), 7.00–6.98 (m, 2H), 3.87–3.83 (dd, *J* = 9.5 Hz, 4 Hz, 1 H), 2.00–1.92 (m, 1H), 1.86–1.73 (m, 2H), 1.70–1.32 (m, 5H); MS *m/z* 198.2 (MH^+^).

**2-Amino-2-(thiophen-2-yl)cyclohexan-1-one (nortiletamine) (35)**. A solution of amino alcohol **34** (0.79 g, 4.0 mmol) in acetone (180 mL) was treated slowly with Jones reagent (2.5 M, 4.0 mmol, 1.6 mL) at room temperature. The reaction was stirred at room temperature for 30 min, filtered and concentrated in vacuo. The residue was diluted with water and neutralised with 2.0 M aq. NaOH solution. The aqueous layer was extracted with Et_2_O (3x 60 mL), dried with MgSO_4_, concentrated in vacuo and purified with flash chromatography EtOAc (100%) to yield **35** (0.66 g, 84%). ^1^HNMR (CDCl_3_) δ 7.29–7.28 (dd, *J* = 5.0 Hz, 1.0 Hz, 1H), 6.97–6.96 (dd, *J =* 5.0 Hz, 3.5 Hz, 1H), 6.82–6.81 (dd, *J* = 3.5 Hz, 1.0 Hz, 1H), 2.74–2.66 (m, 1H), 2.66–2.48 (m, 2H), 2.10–1.90 (m, 2H), 1.90–1.80 (m, 2H), 1.78–1.62 (m, 1H); MS *m/z* 196.2 (MH^+^).

### 4.4. Synthesis of Ketamine Esters (Example) (Scheme 1).

**Methyl 5-((1-(4-chlorophenyl)-2-oxocyclohexyl)amino)pentanoate (15b).** A solution of **22** (0.9 g, 4.03 mmol), methyl 5-bromovalerate (1.02 g, 5.2 mmol), KI (0.23 g, 1.4 mmol), K_2_CO_3_ (1.67 g, 12.0 mmol) in MeCN (20 mL) was heated to a 112 °C in sealed tube for 20 h. The reaction mixture was cooled to room temperature, filtered and the solvent evaporated. The residue was purified by column chromatography on silica gel eluting with EtOAc/hexanes (30–60%) to obtain **15b** (1 g, 74%) as pale yellow oil. This was dissolved in Et_2_O (10 mL) and cooled to 0 °C, HCl in Et_2_O (2M, 4.45 mmol) were added dropwise. The solvent was evaporated under reduced pressure, the residue was taken up in EtOAc (5 mL) and sonicated at room temperature for 2 min. The white precipitate was diluted with EtOAc (5 mL), filtered washed with EtOAc and dried under vacuum to give **15b** as the solid HCl salt. ^1^HNMR (CDCl_3_) δ 7.34–7.28 (m, 2H), 7.19–7.16 (m, 2H), 3.64 (s, 3 H), 2.82–2.72 (m, 1H), 2.48–2.38 (m, 1H), 2.38–2.32 (m, 1H), 2.28–2.18 (m, 3H), 2.08–1.92 (m, 4H), 1.88–1.78 (m, 2H), 1.78–1.64 (m, 2H), 1.48–1.32 (m, 2H); ^13^C (CDCl_3_) δ 210.99, 174.29, 138.32, 133.47, 129.09, 128.67, 69.59, 51.74, 41.90, 39.79, 36.74, 33.88, 30.20, 27.70, 22.76, 22.38; MS *m/z* 338.20 (MH^+^). Calculated for C_18_H_24_ClNO_3_ (MH^+^) 338.15175, found 338.15170.

The other compounds of Table S1 were prepared similarly. See Supplementary Information for details.

### 4.5. Biology

#### 4.5.1. Animals

All animal experiments were conducted at the Ruakura Research Centre, Hamilton, New Zealand, using experimental protocols reviewed and approved by the Ruakura Animal Ethics Committee (ethics ref 12604/13786). Adult female Sprague-Dawley rats of approximately 250–350 g were evaluated in both anaesthetic and analgaesic study protocols. All study agents were delivered by tail vein cannula connected via minibore extension tubing to mechanised infusion pump.

#### 4.5.2. Anaesthetic Assessment Protocol

Following acquisition of baseline physiologic parameters (heart rate, respiratory rate, PWR, and righting reflex (RR)) ketamine or an experimental compound at 10mg/mL were commenced at a rate (weight-adjusted) to deliver 20 mg/kg/min. Dose to loss of righting reflex (LORR see below), and subsequent pedal withdrawal score of 1 (PWR see below) were recorded. Following attaining a PWR of 1, infusion rate was reduced to 6.7 mg/kg/min, then titrated in an up-and-down fashion as required to maintain both dorsal recumbency, and a PWR = 1, to 10 min before cessation. Each study used three rats, with each group of rats also acting as their own ketamine control. Prior odds/evens randomisation determined the order of study drug administration was determined by with a recovery interval of at least three hours afforded between experiments. Records of PWR and RR were made at one-minute intervals throughout, from cessation of infusion to return of righting reflex (RORR), and from cessation of infusion to the animals displaying independent locomotion (walk).

Loss of Righting Reflex (LORR): This is primarily used to assess anaesthetic hypnotic effect. Righting reflex is judged absent when the rat fails to right from a position of dorsal recumbency to a position of sternal recumbency on three attempts performed in rapid succession. Dose to LORR is termed effective potency.

Pedal Withdrawal Reflex (PWR) scoring: Nociceptive testing in animals was conducted via 1 s application of constant pressure (firm digital pressure) over the forepaw of the animal. Pedal withdrawal reflex testing is primarily used to assess analgaesic effect, and responses are graded accordingly: 0, absent; 1, flicker; 2, moderate withdrawal; 3, fast withdrawal; 4, Fast withdrawal with cry/preceding apnoea (modified from [18]).

Behavioural dysfunction scoring: This was undertaken according to the following table. Observations were made over a one minute interval every five minutes from cessation of infusion until return of normal behaviours (total score = 0). A score of 1 was accorded for any positive behavioural aberration for each of four categories (maximal score 4) during wake-up.

Duration of any behavioural aberration was recorded from RORR as follows: score 0 = nil; score1 = 0–120 s; score 2 = 121–300 s; score 3 = 301–600 s; score 4 = 600+ s.

Analgaesic assessment protocol: Animal preparation was in accord with the anaesthetic assessment protocol above. Three rats were used in each study. Following venous cannulation, animals underwent infusion of ketamine or experimental compound at 20 mg/kg over a ten minute interval. A tail flick analgaesia meter (Colombus Instruments, Colombus, Ohio) was then used to determine thermal pain sensitivity. Radiant heat was applied using a shutter-controlled lamp as a heat source focused on a spot located 6–8 cm from the tip of the tail. The intensity of the beam was set at a level producing basal latency times between 2 and 4 s. To prevent thermal tissue injury the cut off time as set at 10 s. A digital response time indicator with a resolution of 0.1s measured the time from initiation of stimulus until tail withdrawal (the flick; TFL).

The TFL response following infusion of control and study drugs was calculated as a percentage of the maximum possible effect (MPE) such that:

%MPE = [TFL (post-drug) – TFL (pre-drug)/10 s – TFL (pre-drug)] × 100%(1)

TFL latency was recorded at 5 min intervals from cessation of study drug infusion (time zero) to 60 min. Individual %MPE-time curves were constructed for each animal, and the area under the curve (AUC) adopted as a composite measure of induced analgaesia (cTFL).

## Figures and Tables

**Table 1:**
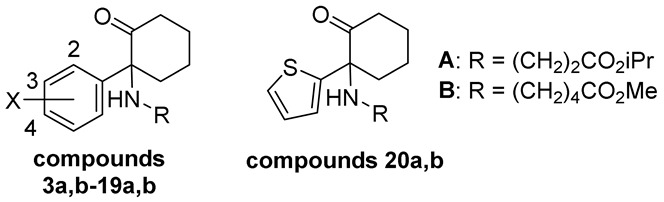
Biological and physiochemical data for esters of ring-substituted ketamine analogues.

No	X	n	R	Sedation		Analgaesia		Rat	NMDA	Behavioural dysfunction	
				LORR ^a^ (mg/kg) (SEM)	RORR ^b^ (sec) (SEM)	potency ^c^ (mg/kg) (SEM)	cTFL ^d^ (sec)	t½ ^e^ (min)	IC_50_ (μM)^f^	Score ^g^ (SEM)	Duration ^h^ (SEM)
**1**	2-Cl	3	Me	21 (4)	1075 (89)	28	671	44.4	0.7	3 (0)	3 (1)
**3a**	H	3	A	>200 (0)	N/A^i^	>200 (0)	0		>1000	0.5 (0.7)	0 (0)
**3b**	H	3	B	40 (5)	78 (22)	45 (5)	0		9.9	1 (0)	2 (0)
**4a**	2-F	3	A	59 (10)	64 (7)	62 (6)	1297		249	1 (0)	1 (0)
**4b**	2-F	2	B	35 (4)	84 (20)	40 (5)	11		3.1	3 (1.4)	4 (0)
**5a^j^**	2-Cl	3	A	33 (10)	83 (33)	37 (11)	1384	3.4	134	2 (1)	1 (0)
**5b^j^**	2-Cl	3	B	34 (12)	99 (28)	44 (17)	374	0.7	3.2	2 (0)	1 (0)
**6a**	2-Me	3	A	>200 (0)	N/A	>200 (0)	393		428	0 (0)	0 (0)
**6b**	2-Me	3	B	37 (2)	85 (14)	43 (3)	91		3.4	1 (0)	2 (1)
**7a**	2-OMe	1	A	>200 (0)	N/A	>200 (0)	313		172	0 (0)	0 (0)
**7b**	2-OMe	2	B	34 (9)	135 (12)	49 (11)	2332		>1000	3 (1)	2 (0)
**8a**	2-CF_3_	2	A	64 (12)	58 (4)	64 (12)	652		290	2 (1)	1 (0)
**8b^#^**	2-CF_3_	2	B	154 (42)	66 (8)	156 (45)	1864		128	1 (0)	1 (0)
**9a**	2-OCF_3_	1	A	62 (0)	65 (0)	62 (0)	80		184	1 (0)	1 (0)
**9b**	2-OCF_3_	2	B	>200 (0)	N/A	>200 (0)	119		121	3 (1)	3 (0)
**10a**	3-Cl	3	A	67 (21)	10 (1)	58 (11)	332		164	1 (0)	1 (0)
**10b**	3-Cl	3	B	51 (6)	114 (46)	61 (4)	54		13	3 (1)	2 (0)
**11a**	3-Me	2	A	38 (4)	108 (39)	50 (5)	63		466	2 (1)	1 (0)
**11b**	3-Me	2	B	39 (6)	137 (32)	43 (6)	104		5.2	2 (1)	1 (0)
**12a**	3-OMe	1	A	79 (0)	10 (0)	83 (0)	284		201	2 (0)	1 (0)
**12b**	3-OMe	2	B	50 (8)	120 (3)	58 (6)	210		5.6	3 (1.4)	4 (0)
**13a^k^**	3-CF_3_	1	A	>200 (0)	N/A	>200 (0)	208		229	0 (0)	0 (0)
**13b^#^**	3-CF_3_	2	B	>200 (0)	N/A	>200 (0)	8		172	0 (0)	0 (0)
**14a**	3-OCF_3_	2	A	>200 (0)	N/A	>200 (0)	274		211	0 (0)	0 (0)
**14b**	3-OCF_3_	3	B	>200 (0)	N/A	>200 (0)	444		57	0 (0)	0 (0)
**15a**	4-Cl	3	A	99 (43)	15 (9)	>200 (0)	491		129	2 (1)	1 (0)
**15b**	4-Cl	3	B	89 (40)	82 (40)	100 (23)	576		101	3 (1)	3 (0)
**16a**	4-Me	1	A	47 (0)	10 (0)	52 (0)	650		161	1 (0)	1 (0)
**16b**	4-Me	2	B	50 (6)	75 (11)	55 (9)	0		23	1 (0)	0 (0)
**17a**	4-OMe	2	A	>200 (0)	N/A	>200 (0)	167		396	0 (0)	0 (0)
**17b**	4-OMe	1	B	Seizure			551		94.1	3 (0)	3 (0)
**18a^#^**	4-CF_3_	1	A	>200 (0)	N/A	>200 (0)	412		438	0 (0)	0 (0)
**18b^#^**	4-CF_3_	2	B	>200 (0)	N/A	>200 (0)	37		470	0 (0)	0 (0)
**19a**	4-OCF_3_	1	A	>200 (0)	N/A	>200 (0)	166		314	0 (0)	0 (0)
**19b**	4-OCF_3_	1	B	>200 (0)	N/A	>200 (0)	194		597	0 (0)	0 (0)
**20a**	thioph	3	A	>200 (0)	N/A	>200 (0)	362		157	1 (1)	1 (0)
**20b**	thioph	2	B	52 (6)	10 (2)	74 (7)	196		8.9	4 (0)	1 (0)

^a^ LORR: minimal dose (mg/kg) for loss of righting reflex (measure of sedative potency); ^b^ RORR: time (sec) for return of righting reflex after infusion stopped; ^c^ minimal dose (mg/kg) for loss of pedal withdrawal reflex (measure of analgaesic potency); ^d^ cTFL (composite tail flick latency, a measure of analgaesic magnitude/duration); ^e^ Drug half-life in rats (see ref 10 for method); ^f^ NMDA; IC_50_ (µM) for inhibition of the *N*-methyl-D-aspartate receptor; conducted by Eurofins Eurofins Panlabs Taiwan; ^g^ Severity of behavioural dysfunction on recovery (see biology methods); ^h^ Duration of behavioural dysfunction (see biology methods); ^i^ N/A; animals failed to demonstrate LORR (sedation)/PWR of 1 (analgaesia) [9].

**Table 2:** Behavioural dysfunction score (score 1 for any behavioural aberration in each category to maximum score 4).

(1) General	Exophthalmos
	Increased respiration
	Decreased respiration
(2) Motor	Hunched posturing
	Subdued
	Increased/decreased body tone
	Increased/decreased locomotor activity
(3) Reactivity	Increased irritability on gentle handling
	Decreased irritability on gentle handling
	Rearing
(4) Stereotypical behaviours	Head weaving
	Ataxia/decreased co-ordination
	Splayed hind legs

## Supplementary Materials

The following are available online, Syntheses and characterisation of the compounds of Table S1.

## Abbreviations

DCM	Dichloromethane
LORR	Loss of righting reflex
NMDA	*N*-methyl-D-aspartate
PWR	Pedal withdrawal reflex score
TFAA	Trifluoroacetic acid anhydride.
