# Supplementary material for: Structure-Activity Relationships for the Anaesthetic and Analgaesic Properties of Aromatic Ring-Substituted Ketamine Esters
Source: Molecules. 2020 Jun 26;25(12):2950. doi: 10.3390/molecules25122950 (PMC7356787; doi:10.3390/molecules25122950)
Supplement: Supplementary file 1 [file molecules-25-02950-s001.pdf]

molecules

MDPI

Supplementary Materials

Structure-Activity Relationships for the Anaesthetic and Analgaesic Properties of Aromatic Ring-Substituted Ketamine Esters

Ivaylo V. Dimitrov [1], Martyn G. Harvey [2], Logan J. Voss [2], James W. Sleigh [2] and Michael J. Bickerdike [3], William A. Denny [1,*]

[1] Auckland Cancer Society Research Centre, School of Medical Sciences; i.dimitrov@auckland.ac.nz
[2] Waikato Clinical School, University of Auckland, Private Bag 92019, Auckland 1142, New Zealand; Martyn.Harvey@waikatodhb.health.nz (M.G.H.); Logan.Voss@waikatodhb.health.nz (L.J.V.); Jamie.Sleigh@waikatodhb.health.nz (J.W.S.)
[3] Kea Therapeutics Ltd, Auckland, New Zealand; Mike@keatx.com
* Correspondence: b.denny@auckland.ac.nz

Supplementary Information (syntheses and characterisation of the compounds of Table 1)

Preparation of the other esters of Table 1 (see example of 15b in text)

Isopropyl 3-((2-oxo-1-phenylcyclohexyl)amino)propanoate (3a). Reaction of amine **21** (0.14 g, 0.74 mmol) with isopropyl 3-bromopropanoate (1.15 g, 5.90 mmol) gave **3a** (0.1 g, 45%), which was converted as above to the solid HCl salt. ^{1}HNMR ($CDCl_3$) δ 7.50–7.46 (m, 1H), 7.39–7.30 (m, 3H), 7.29–7.24 (m, 1H), 5.04–4.93 (m, 1H), 2.84–2.16 (m, 4H), 2.08–1.92 (m, 4H), 1.92–1.62 (m, 4H), 1.24–1.21 (d, *J* = 6.40 Hz, 6 H); ^{13}C ($CDCl_3$) δ 208.26, 171.38, 137.11, 129.76, 128.94, 128.62, 127.36, 72.30, 68.03, 41.64, 38.66, 37.36, 35.92, 28.44, 22.42, 21.99; *m*/*z* 304.30 (MH$^+$), Calculated for $C_{18}H_{25}NO_3$ (MH$^+$) 304.1907, found 304.1915.

Isopropyl 3-((1-(2-fluorophenyl)-2-oxocyclohexyl)amino)propanoate (4a). Similar reaction of **30b** (0.18 g, 0.89 mmol) with isopropyl 3-bromopropanoate (2.09 g, 10.70 mmol) gave (**4a**) (0.37 g, 70%), which was converted as above to the HCl salt. ^{1}HNMR ($CDCl_3$) δ 7.46–7.43 (td, *J* = 7.74 Hz, 1.70 Hz, 1H), 7.32–7.28 (m, 1H), 7.22–7.18 (td, *J* = 7.60 Hz, 1.30 Hz, 1H), 7.09–7.04 (ddd, *J* = 8.10 Hz, 3.42 Hz, 1.20 Hz, 1H), 5.05–4.98 (m, 1H), 2.65–2.62 (m, 1H), 2.58–2.53 (m, 2H), 2.46–2.44 (m, 4H), 1.94–1.72 (m, 5H), 1.26–1.24 (d, *J* = 9.44 Hz, 6H); ^{13}C ($CDCl_3$) δ 209.22, 172.54, 159.92, 129.55- 128.64, 124.36, 116.57, 116.34, 68.05, 60.60, 39.42, 38.96, 38.37, 35.61, 27.97, 22.04, 21.96; MS *m*/*z* 322.20 (MH$^+$). Calculated for $C_{18}H_{24}FNO_3$ (MH$^+$) 322.1813, found 322.18225.

Isopropyl 3-((2-oxo-1-(*o*-tolyl)cyclohexyl)amino)propanoate (6a). Similar reaction of amine **30e** (0.7 g, 3.44 mmol) with isopropyl 3-bromopropanoate (4.03 g, 21.0 mmol) gave **6a** (0.8 g, 74%), which was converted as above to the solid HCl salt. ^{1}HNMR ($CDCl_3$) δ 7.54–7.52 (dd, *J* = 5.76 Hz, 3.2 Hz, 1H), 7.28–7.18 (m, 2H), 7.16–7.12 (m, 1H), 5.05–4.94 (m, 1H), 2.68–2.54 (m, 1H), 2.42–2.30 (m, 2 H), 2.24–2.18 (m, 1H), 2.06–1.92 (m, 2H), 1.86–1.72 (m, 5 H), 1.70–1.56 (m, 1H), 1.25–1.21 (d, *J* = 6.28 Hz, 6 H); ^{13}C ($CDCl_3$) δ 212.32, 172.63, 138.06, 137.30, 132.72, 128.74, 127.96, 125.80, 70.62, 67.92, 40.51, 39.79, 37.76, 35.86, 29.72, 22.62, 22.04, 21.16; MS *m*/*z* 318.30 (MH$^+$), Calculated for $C_{19}H_{27}NO_3$ (MH$^+$) 318.2064, found 318.2069.

Isopropyl 3-((1-(2-methoxyphenyl)-2-oxocyclohexyl)amino)propanoate (7a). Similar reaction of amine **30h** (0.2 g, 0.90 mmol) with isopropyl 3-bromopropanoate (1.6 g, 8.2 mmol) gave **7a** (0.16 g, 53%), which was converted as above to the solid HCl salt. ^{1}HNMR ($CDCl_3$) δ 7.47–7.45 (dd, *J* = 7.72 Hz, 1.48 Hz, 1H), 7.35–7.30 (td, *J* = 8.12 Hz, 1.6 Hz, 1H), 7.07–7.03 (td, *J* = 7.57 Hz, 1.08 Hz, 1H), 6.93–6.91 (dd, *J* = 8.17 Hz, 0.92 Hz, 1H), 5.02–4.96 (m, 1H), 3.75 (s, 3H), 2.84–2.82 (m, 1H), 2.55–2.52 (m, 1 H), 2.42–2.41 (m, 4H), 1.74–1.68 (m, 6 H), 1.23–1.21 (d, *J* = 4.84 Hz, 6H), ^{13}C ($CDCl_3$) δ 210.42, 172.32, 157.19, 129.05, 128.20, 120.82, 111.70, 68.19, 37.69, 55.19, 39.21, 38.64, 38.17, 35.36, 28.77, 22.05, 21.80; MS *m*/*z* 334.30 (MH$^+$), Calculated for $C_{19}H_{27}NO_4$ (MH$^+$) 334.2026, found 334.2029.

Isopropyl 3-((2-oxo-1-(2-(trifluoromethyl)phenyl)cyclohexyl)amino)propanoate (8a). Similar reaction of amine **30k** (0.25 g, 0.72 mmol) with isopropyl 3-bromopropanoate (2.20 g, 12.00 mmol) gave **8a** (0.36 g, 72%), which was converted as above to the HCl salt. ^{1}HNMR ($CDCl_3$) δ 7.89- 7.87 (d, *J* = 8.00 Hz, 1H), 7.66–7.64 (d, *J* = 7.88 Hz, 1H), 7.54–7.50 (t, *J* = 7.88 Hz, 1H), 7.40–7.37 (t, *J* = 7.60 Hz, 1H), 5.08–5.00 (m, 1H), 2.56–2.46 (m, 5H), 2.32–2.27 (m, 3H), 1.95–1.83 (m, 4H), 1.26–1.24 (d, *J* = 7.04 Hz, 6H), ^{13}C ($CDCl_3$) δ 205.12, 172.96, 139.90, 131.89, 129.30, 128.98, 128.28, 127.62, 68.33, 68.01, 40.88, 38.28, 38.25, 35.00, 33.96, 24.78, 22.12, 21.28; MS *m/z* 372.30 (MH^+), Calculated for $C_{19}H_{24}F_3NO_3$ (MH^+) 372.1781 found 372.1791.

Isopropyl 3-((2-oxo-1-(2-(trifluoromethoxy)phenyl)cyclohexyl)amino)propanoate (9a). Similar reaction of amine **30n** (0.15 g, 0.55 mmol) with isopropyl 3-bromopropanoate (1.07 g, 5.50 mmol) gave **9a** (0.15 g 70%), which was converted as above to the HCl salt. ^{1}HNMR δ 7.95–7.93 (d, *J* = 7.96 Hz, 1H), 7.76–7.72 (t, *J* = 7.80 Hz, 1H), 7.61–7.58 (t, *J* = 7.72 Hz, 1H), 7.54–7.52 (d, *J* = 8.40 Hz, 1H), 5.01–4.95 (m, 1H), 3.02–2.99 (m, 1H), 2.84–2.82 (m, 1H), 2.70–2.67 (m, 1H), 2.55–2.43 (m, 2H), 2.14–2.12 (m, 4H), 2.00–1.94 (m, 2H), 1.84–1.73 (m, 1H), 1.21–1.19 (dd, *J* = 6.24 Hz, 2.12 Hz, 6H), ^{13}C ($CDCl_3$) δ 210.52, 174.36, 149.87, 134.62, 132.58, 128.65, 127.65, 122.95, 70.53, 67.06, 40.10, 39.56, 26.90, 30.38, 24.36, 22.96, 22.03; MS *m/z* 388.20 (MH^+), Calculated for $C_{19}H_{25}F_3NO_4$ (MH^+) 388.1730 found 388.1730.

Isopropyl 3-((1-(3-chlorophenyl)-2-oxocyclohexyl)amino)propanoate (10a). Similar reaction of amine **30c** (0.6 g, 2.69 mmol) with isopropyl 3-bromopropanoate (4.2 g, 21.0 mmol) gave **10a** (1.2 g, 80%), which was converted as above to the solid HCl salt. ^{1}HNMR ($CDCl_3$) δ 7.32–7.26 (m, 2H), 7.26–7.20 (m, 1H), 7.18–7.12 (dt, *J* = 5.96 Hz, 1.52 Hz, 1H), 5.08–4.94 (m, 1H), 2.52–2.48 (m, 2H), 2.42–2.34 (t, *J* = 5.92 Hz, 2H), 2.32–2.22 (m, 2H), 1.98–1.82 (m, 3H), 1.80–1.50 (m, 4H), 1.22–1.21 (d, *J* = 6.28 Hz, 6 H); ^{13}C ($CDCl_3$) δ 210.33, 172.38, 142.01, 135.01, 130.24, 128.34, 127.80, 125.60, 69.42, 68.03, 39.78, 38.11, 37.25, 35.70, 27.60, 22.32, 22.05; MS *m/z* 338.20 (MH^+), Calculated for $C_{18}H_{24}ClNO_3$ (MH^+) 338.1517, found 338.1515.

Isopropyl 3-((2-oxo-1-(*m*-tolyl)cyclohexyl)amino)propanoate (11a). Similar reaction of amine **30f** (0.16 g, 0.78 mmol) with isopropyl 3-bromopropanoate (1.40 g, 7.10 mmol) gave **11a** (0.6 g, 66%), which was converted as above to the solid HCl salt. ^{1}HNMR ($CDCl_3$) δ 2.27–7.23 (m, 1H), 7.10–7.02 (m, 3H), 5.04–4.92 (m, 1H), 2.88–2.84 (m, 1H), 2.64–2.54 (m, 1H), 2.44–2.38 (m, 1H), 2.34–2.22 (m, 3H), 2.32 (s, 3H), 2.00–1.89 (m, 2H), 1.88–1.67 (m, 4H), 1.22–1.18 (d, *J* = 4.16 Hz, 6H); 13C (CDCl3) δ 211.32, 172.40, 139.24, 138.70, 128.86, 128.47, 127.88, 124.29, 69.70, 67.87, 39.88, 38.18, 36.56, 35.86, 27.86, 22.53, 22.53, 21.84, 21.26; MS *m/z* 318.30 (MH+), Calculated for $C_{19}H_{27}NO_3$ (MH^+) 318.2064, found 318.2069.

Isopropyl 3-((1-(3-methoxyphenyl)-2-oxocyclohexyl)amino)propanoate (12a). Similar reaction of amine **30i** (0.26 g, 1.18 mmol) with isopropyl 3-bromopropanoate (1.3 g, 7.12 mmol) gave **12a** (0.28 g, 72%), which was converted as above to the solid HCl salt. ^{1}HNMR ($CDCl_3$) δ 7.36–7.28 (m, 1H), 6.84–6.80 (m, 3H), 5.00–4.94 (m, 1H), 3.80 (s, 3H), 2.86–2.80 (m, 1H), 2.53–2.48 (m, 1H), 2.42- 2.38 (m, 1H), 2.38- 2.22 (m, 2H), 1.98- 1.91 (m, 2H), 1.88–1.68 (m, 5H), 1.22–1.10 (d, *J* = 9.44 Hz, 6H); ^{13}C ($CDCl_3$) δ 210.98, 172.39, 160.22, 141.04, 130.02, 119.63, 113.44, 112.58, 69.62, 67.88, 55.46, 39.84, 38.13, 36.69, 35.85, 27.76, 22.51, 22.04; MS m/z 334.20 (MH^+), Calculated $C_{19}H_{27}NO_4$ for (MH^+) 334.2013, found 334.2018.

Isopropyl 3-((2-oxo-1-(3-(trifluoromethyl)phenyl)cyclohexyl)amino)propanoate (13a). Similar reaction of amine **30l** (0.26 g, 1.00 mmol) with isopropyl 3-bromopropanoate (2.30 g, 12.00 mmol) gave **13a** (0.25, 68%) which was converted as above to the HCl salt. ^{1}HNMR ($CDCl_3$) δ 7.55–7.53 (m, 1H), 7.53–7.44 (m, 3H), 5.01–4.97 (m, 1H), 2.70–2.68 (m, 1H), 2.58–2.49 (m, 2H), 2.44–2.39 (m, 2H), 2.32–2.28 (m, 2H), 1.96–1.80 (m, 2H), 1.76–1.58 (m, 3H), 1.25–1.22 (d, *J* = 6.24 Hz, 6H), ^{13}C ($CDCl_3$) δ 210.24, 172.42, 141.22, 131.39, 130.98, 129.39, 124.55, 124.05, 123.98, 69.47, 68.11, 39.72, 38.14, 37.99, 35.56, 27.51, 22.19, 22.04; MS *m/z* 372.20 (MH^+), Calculated for $C_{19}H_{24}F_3NO_3$ (MH^+) 372.1793 found 372.1794.

Isopropyl 3-((2-oxo-1-(3-(trifluoromethoxy)phenyl)cyclohexyl)amino)propanoate (14a). Similar reaction of amine **30o** (0.26 g, 0.95 mmol) with isopropyl 3-bromopropanoate (2.20 g, 11.40 mmol) gave **14a** (0.27 g 76%), which was converted as above to the HCl salt. ^{1}HNMR δ 7.41, 7.37 (t, J = 7.92 Hz, 1H), 7.19–7.12 (m, 3H), 5.02–4.96 (m, 1H), 2.70–2.62 (m, 1H), 2.53–2.49 (m, 2H), 2.40–2.37 (t, J = 6.56 Hz, 2H), 2.32–2.27 (m, 2H), 1.94–1.18 (m, 5H), 1.24–1.23 (d, J = 6.24 Hz, 6H), ^{13}C ($CDCl_3$) δ 210.20,

172.39, 149.82, 142.54, 130.26,125.88, 125.87, 120.11, 119.98, 69.38, 38.06, 39.70, 38.12, 37.72, 35.64, 27.51, 22.03, 22.02; MS *m/z* 388.20 (MH+), Calculated for $C_{19}H_{24}F_3NO_4$ (MH+) 388.1755 found 388.1756.

Isopropyl 3-((1-(4-chlorophenyl)-2-oxocyclohexyl)amino)propanoate (15a). Similar reaction of amine **22** (1.0 g, 4.48 mmol) with isopropyl 3-bromopropanoate gave **15a** (1.2 g, 80%) as pale yellow oil, which was converted as above to the solid HCl salt.^{1}HNMR ($CDCl_3$) δ 7.36–7.26 (m, 2H), 7.22–7.19 (m, 2H), 5.01–4.95 (m, 1H), 2.78–2.66 (m, 1H), 2.52–2.44 (m, 2H), 2.38–2.33 (t, *J* = 7.52Hz, 2H), 2.32–2.22 (m, 2H), 1.98–1.58 (m, 5H), 1.22–1.20 (d, *J* = 6.28 Hz, 6H); ^{13}C ($CDCl_3$) δ 210.64, 172.38, 138.26, 133.54, 129.26, 128.79, 68.01, 41.20, 38.55, 37.88, 35.68, 27.66, 22.50, 22.33, 22.04; MS *m/z* 338.20 (MH^+), Calculated for $C_{18}H_{24}ClNO_3$ (MH^+) 338.1518, found 338.1515.

Isopropyl 3-((2-oxo-1-(*p*-tolyl)cyclohexyl)amino)propanoate (16a). Similar reaction of amine **30g** (0.23 g, 1.13 mmol) with isopropyl 3-bromopropanoate (2.20 g, 11.3 mmol) gave **16a** (0.2 g, 56%), which was converted as above to the solid HCl salt. ^{1}HNMR ($CDCl_3$) δ 7.18–7.15 (d, *J* = 8.04 Hz, 2H), 7.11–7.09 (d, *J* = 8.2 Hz, 2H), 5.00–4.88 (m, 1H), 2.84–2.82 (m, 1H), 2.52–2.46 (m, 1H), 2.44–2.22 (m, 5H), 2.34 (s, 3H), 1.98–1.88 (m, 1H), 1.88–1.72 (m, 4H), 1.21–1.19 (d, *J* = 6.24 Hz, 6H); ^{13}C ($CDCl_3$) δ 211.16, 172.20, 137.18, 136.09, 129.52, 126.96, 69.27, 67.64, 39.56, 37.92, 36.46, 35.64, 27.64, 22.29, 21.82, 20.98; MS *m/z* 318.20 (MH^+), Calculated for $C_{19}H_{27}NO_3$ (MH^+) 318.2064, found 318.2080.

Isopropyl 3-((1-(4-methoxyphenyl)-2-oxocyclohexyl)amino)propanoate (17a). Similar reaction of amine **30j** (0.10 g, 0.45 mmol) with isopropyl 3-bromopropanoate (1.06 g, 5.47 mmol) gave **17a** (0.1 g, 66%), which was converted as above to the solid HCl salt. ^{1}HNMR ($CDCl_3$) δ 7.18–7.16 (d, *J* = 8.88 Hz, 2H), 6.91–6.88 (d, *J* = 8.88 Hz, 2H), 5.00–4.94 (m, 1H), 3.81 (s, 3H), 2.84–2.80 (m, 1 H), 2.38–2.28 (m, 4H), 1.85–1.73 (m, 6H), 1.21–1.19 (d, J = 6.28 Hz); ^{13}C ($CDCl_3$) δ 211.21, 172.44, 159.08, 130.98, 128.55, 114.18, 69.36, 67.98, 55.47, 39.69, 38.20, 36.74, 35.65, 27.83, 22.50, 22.05; MS *m/z* 334.20 (MH^+), Calculated for $C_{19}H_{27}NO_4$ (MH^+) 334.2013, found 334.2018.

Isopropyl 3-((2-oxo-1-(4-(trifluoromethyl)phenyl)cyclohexyl)amino)propanoate (18a). Similar reaction of amine **30m** (0.18 g, 0.70 mmol) with isopropyl 3-bromopropanoate (1.64 g, 8.40 mmol) gave **18a** (0.18 g 69%) which was converted as above to the HCl salt. ^{1}HNMR ($CDCl_3$) δ 7.63- 7.61 (d, *J* = 8.20 Hz, 2H), 7.41–7.7.39 (d, *J* = 8.10 Hz, 2H), 5.00–4.97 (m, 1H), 2.60–2.40 (m, 2H), 2.40–2.20 (m, 5H), 1.96–1.82 (m, 3H). 1.79–1.60 (m, 2H), 1.26–1.24 (d, *J* = 8.22 Hz, 6H), ^{13}C ($CDCl_3$) δ 210.22, 172.42, 144.03, 129.35, 127.79, 125.83, 69.50, 68.11, 39.76, 38.64, 38.10, 37.70, 36.84, 35.53, 27.53, 22.20; MS *m/z* 372.20 (MH+), Calculated for $C_{19}H_{24}F_3NO_3$ (MH+) 372.1781 found 372.1790.

Isopropyl 3-((2-oxo-1-(4-(trifluoromethoxy)phenyl)cyclohexyl)amino)propanoate (19a). Similar reaction of amine **30p** (0.48 g, 1.76 mmol) with isopropyl 3-bromopropanoate (3.42 g, 18.0 mmol) gave **19a** (0.50 g, 73%), which was converted as above to the HCl salt. ^{1}HNMR δ 7.31–7.29 (d, *J* = 6.72 Hz, 2H), 7.22–7.19 (d, *J* = 8.04 Hz, 2H), 5.06–4.94 (m, 1H), 2.72–2.65 (m, 1H), 2.53–2.47 (m, 2H), 2.40–2.36 (t, *J* = 6.52 Hz, 2H), 2.33–2.26 (m, 2H), 1.94–1.68 (m, 5H), 1.26–1.24 (d, J = 8.22 Hz, 6H), ^{13}C ($CDCl_3$) δ 21057, 172.42, 148.54, 138.52, 128.87, 121.14, 69.21, 68.37, 39.73, 38.12, 37.73, 35.66, 27.60, 22.27, 22.04; MS *m/z* 388.20 (MH+), Calculated for $C_{19}H_{24}F_3NO_4$ (MH^+) 388.1755 found 388.1752.

Isopropyl 3-((2-oxo-1-(thiophen-2-yl)cyclohexyl)amino)propanoate (20a). Similar reaction of nortiletamine (**35**) (0.16 g, 0.82 mmol) with isopropyl 3-bromopropanoate (0.96 g, 4.90 mmol) gave **20a** (0.1 g, 40%), which was converted as above to the solid HCl salt. ^{1}HNMR ($CDCl_3$) δ 7.36–7.28 (m, 1H), 7.00–6.97 (m, 2H), 6.92–6.90 (dd, *J* = 3.56 Hz, 1.12 Hz, 1H), 5.04–4.94 (m, 1H), 2.88–2.84 (m, 1H), 2.66–2.40 (m, 4H), 2.04–1.98 (m, 1H), 1.86–1.78 (m, 4H), 1.23–1.21 (d, *J* = 6.28 Hz, 6H), ^{13}C ($CDCl_3$) δ 209.52, 172.41, 147.98, 127.13, 126.62, 125.32, 67.97, 67.64, 40.68, 39.54, 39.18, 38.37, 35.73, 27.24, 22.06; MS *m/z* 310.20 (MH^+), Calculated for $C_{16}H_{23}NO_3S$ (MH^+) 310.1471, found 310.1476.

Methyl 5-((2-oxo-1-phenylcyclohexyl)amino)pentanoate (3b). Similar reaction of amine **21** (0.17 g, 0.90 mmol) with methyl 5-bromovalerate (0.22 g, 1.1 mmol) gave **3b** (0.2 g, 74%), which was converted as above to the solid HCl salt. ^{1}HNMR ($CDCl_3$) δ 7.38–7.34 (m, 2H), 7.29–7.21 (m, 3H), 3.62 (s, 3H), 2.88–2.84 (m, 1H), 2.42–2.38 (m, 1H), 2.36–2.20 (m, 3H), 2.06–1.90 (m, 3H), 1.88–1.68 (m, 4H), 1.60–1.50 (m, 2H), 1.48–1.30 (m, 2H); ^{13}C ($CDCl_3$) δ 211.58, 174.22, 139.54, 129.00, 127.63, 127.20, 69.82, 51.63,

41.96, 39.92, 36.38, 34.02, 30.36, 27.88, 22.84, 22.52; MS *m/z* 304.30 (MH^+), Calculated for $C_{18}H_{25}NO_3$ (MH^+) 304.1907, found 304.1909.

Methyl 5-((1-(2-fluorophenyl)-2-oxocyclohexyl)amino)pentanoate (4b). Similar reaction of amine **30b** (0.14 g, 0.68 mmol) with methyl 5-bromovalerate (0.17 g, 0.88 mmol) gave **4b** (0.17 g, 77%), which was converted as above to the solid HCl salt. ^{1}HNMR ($CDCl_3$) δ 7.40–7.37 (td, *J* = 7.76 Hz, 1.72 Hz, 1H), 7.30–7.26 (m, 1), 7.20–7.16 (td, *J* = 7.64 Hz, 1.32 Hz, 1H), 7.07–7.02 (ddd, *J* = 8.12 Hz, 3.40 Hz, 1.24 Hz, 1H), 3.64 (s, 3H), 2.58–2.54 (m, 1H), 2.37–2.34 (m, 2H), 2.26–2.22 (m, 3H), 1.93–1.68 (m, 8H), 1.62–1.55 (m, 2H), ^{13}C ($CDCl_3$) δ 210.05, 174.35, 160.00, 129.61, 128.82, 124.42, 116.69, 116.35, 69.64, 51.76, 42.25, 39.57, 38.99, 34.02, 30.26, 28.48, 22.90, 22.16; MS *m/z* 322.20 (MH^+), Calculated for $C_{18}H_{24}FNO_3$ (MH^+) 322.1813, found 322.1830.

Methyl 5-((2-oxo-1-(*o*-tolyl)cyclohexyl)amino)pentanoate (6b). Similar reaction of amine **30e** (0.5 g, 2.40 mmol) with methyl 5-bromovalerate (0.64 g, 3.20 mmol) gave **6b** (0.6 g, 76%), which was converted as above to the solid HCl salt. ^{1}HNMR ($CDCl_3$) δ 7.46–7.44 (dd, *J* = 7.64 Hz, 1.68 Hz, 1H), 7.26–7.17 (m, 2H), 7.16–7.12 (dd, *J* = 7.16 Hz, 1.8 Hz), 3.62 (s, 3H), 3.05–3.01 (m, 1H), 2.44–2.34 (m, 3H), 2.18–2.12 (t, *J* = 8.28 Hz, 4H), 2.10–1.92 (m, 2H), 1.82–1.72 (m, 3H), 1.62–1.49 (m, 2H), 1.32–1.42 (m, 1H); ^{13}C ($CDCl_3$) δ 213.26, 174.30 138.16, 137.46, 132.66, 127.98, 127.60, 125.66, 70.79, 51.60, 41.10, 40.72, 39.94, 33.90, 30.38, 30.18, 22.76, 22.68; MS *m/z* 318.30 (MH^+), Calculated for $C_{19}H_{27}NO_3$ (MH^+) 318.2064, found 318.2062.

Methyl 5-((1-(2-methoxyphenyl)-2-oxocyclohexyl)amino)pentanoate (7b). Similar reaction of amine **30h** (0.22 g, 1.02 mmol) with methyl 5-bromovalerate (0.22 g, 1.33 mmol) gave **7b** (0.24 g, 70%), which was converted as above to the solid HCl salt. ^{1}HNMR ($CDCl_3$) δ 7.42–7.39 (dd, *J* = 7.68 Hz, 1.68 Hz, 1H), 7.29–7.27 (td, *J* = 7.48 Hz, 1.6 Hz, 1H), 7.04–7.02 (td, *J* = 7.6 Hz, 1.16 Hz, 1H), 6.89–6.88 (dd, *J* = 8.29 Hz, 0.92 Hz, 1H), 3.72 (s, 3H), 3.62 (s, 3H), 2.81–2.78 (m 1H), 2.36–2.34 (m, 1H), 2.24–2.18 (m, 2H), 2.16–2.12 (m, 1H), 1.92–1.90 (m, 1H), 1.69–1.67 (m, 6H), 1.55–1.53 (m, 2H), 1.27–1.24 (m, 2H); ^{13}C ($CDCl_3$) δ 211.58, 174.34, 157.43, 129.03 128.76, 128.44, 120.89, 111.82, 68.38, 55.34, 51.64, 42.07, 39.59, 39.17, 34.06, 30.30, 29.36, 22.86, 22.40; MS *m/z* 334.20 (MH^+), Calculated for $C_{19}H_{27}NO_4$ (MH^+) 334.2013, found 334.2017.

Methyl 5-((2-oxo-1-(2-(trifluoromethyl)phenyl)cyclohexyl)amino)pentanoate (8b). Similar reaction of amine **30k** (0.27 g, 1.05mmol) with methyl 5-bromovalerate (0.27 g, 1.40 mmol) gave **8b** (0.27 g, 72%), which was converted as above to the HCl salt. ^{1}HNMR ($CDCl_3$) δ 7.83–7.82 (d, *J* = 8.04 Hz, 1H), 7.66–7.64 (dd, *J* = 7.89 Hz, 1.28 Hz, 1H), 7.54–7.52 (t, *J* = 7.88 Hz, 1H), 7.41–7.37 (t, *J* = 7.60 Hz, 1H), 3.67 (s, 3H), 2.58–2.44 (m, 2H), 2.32–2.22 (m, 4H), 2.09–1.78 (m, 5H), 1.70–1.44 (m, 5H), ^{13}C ($CDCl_3$) δ 205.37, 174.22, 140.05, 131.74, 129.03, 128.88, 128.49, 127.62, 68.89, 51.75, 42.25, 40.81, 38.34, 34.02, 30.23, 25.21, 22.71, 21.38; MS *m/z* 372.30 (MH^+), Calculated for $C_{19}H_{24}F_3NO_3$ (MH^+) 372.1793 found 372.1791.

Methyl 5-((2-oxo-1-(2-(trifluoromethoxy)phenyl)cyclohexyl)amino)pentanoate (9b). Similar reaction of amine **30n** (0.11 g, 0.40 mmol) with methyl 5-bromovalerate (0.13 g, 0.64 mmol) gave **9b** (0.12 g, 75%), which was converted as above to the HCl salt. ^{1}HNMR ($CDCl_3$) δ 7.53–7.52 (dd, *J* = 7.52 Hz, 1.6 Hz, 1H), 7.34–7.26 (m, 3H), 3.64 (s, 3H), 2.50–2.32 (m, 4H), 2.27–2.23 (t, *J* = 7.33 Hz, 2H), 1.94–1.86 (m, 4H), 1.80–1.70 (m, 4H), 1.6–1.52 (m, 2H), ^{13}C ($CDCl_3$) δ 208.59, 174.36, 147.98, 134.08, 129.89, 129.15, 128.94, 125.96, 118.59, 69.65, 51.77, 42.09, 39.37, 39.03, 34.02, 30.29, 30.29, 27.94, 22.76, 21.67; MS *m/z* 388.20 (MH^+), Calculated for $C_{19}H_{25}F_3NO_4$ (MH^+) 388.1730 found 388.1740.

Methyl 5-((1-(3-chlorophenyl)-2-oxocyclohexyl)amino)pentanoate (10b). Similar reaction of amine **30c** (0.6 g, 2.69 mmol) with methyl 5-bromovalerate (0.65 g, 3.36 mmol), gave **10b** (0.77 g, 84%), which was converted as above to the solid HCl salt. ^{1}HNMR ($CDCl_3$) δ 7.32–7.27 (m, 1H), 7.26–7.24 (m, 2H), 7.11–7.09 (dt, *J* = 7.44 Hz, 1.6 Hz, 1H), 3.64 (s, 3H), 2.48–2.42 (m, 1H), 2.32–2.22 (m, 4H), 2.03–1.93 (m, 2H), 1.88–1.68 (m, 5H), 1.63–1.53 (m, 2H), 1.48–1.32 (m, 2H); ^{13}C ($CDCl_3$) δ 210.72, 174.18, 142.08, 135.04, 130.26, 127.86, 127.38, 125.47, 68.64, 51.66, 41.94, 39.88, 36.64, 34.00, 32.24, 27.68, 22.80, 22.41; MS *m/z* 338.20 (MH^+), Calculated for $C_{18}H_{24}ClNO_3$ (MH^+) 338.1518, found 338.1513.

Methyl 5-((2-oxo-1-(*m*-tolyl)cyclohexyl)amino)pentanoate (11b). Similar reaction of amine **30f** (0.19 g, 0.94 mmol) with methyl 5-bromovalerate (0.23 g, 1.2 mmol) gave **11b** (0.2 g, 68%), which was converted as above to the solid HCl salt. ^{1}HNMR ($CDCl_3$) δ 7.26–7.23(m, 1H), 7.09–7.07 (d, *J* = 7.48 Hz, 1H), 7.04–7.00 (m, 2H), 3.62 (s, 3H), 2.88–2.84 (m, 1H), 2.42–2.30 (m, 2H), 2.34 (s, 3H), 2.22–2.20 (t, *J* = 7.64 Hz, 3H), 2.04–1.92 (m, 2H), 1.88–1.62 (m, 6H), 1.62–1.52 (m, 2H); ^{13}C ($CDCl_3$) δ 211.76, 174.26, 139.46, 138.72, 128.82, 128.41, 127.80, 124.23, 69.80, 51.65, 42.00, 39.99, 36.28, 34.06, 30.30, 27.90, 22.88, 22.60, 21.84; MS *m*/*z* 318.20 (MH+), Calculated for $C_{19}H_{27}NO_3$ (MH^+) 318.2064, found 318.2063.

Methyl 5-((1-(3-methoxyphenyl)-2-oxocyclohexyl)amino)pentanoate (12b). Similar reaction of amine **30i** ((0.16 g, 0.73 mmol) and methyl 5-bromovalerate (0.18 g, 0.95 mmol) gave **12b** (0.18 g, 76%), which was converted as above to the solid HCl salt. ^{1}HNMR ($CDCl_3$) δ 7.30–7.26 (m, 1H), 6.82–6.76 (m, 3H), 3.80 (s, 3H), 3.62 (s, 3H), 2.86 (m, 1H), 2.39–2.20 (m, 5 H), 2.06–1.90 (m, 2H), 1.90–1.68 (m, 6H), 1.62–1.52 (m, 2H), 1.48–1.32 (m, 2H); ^{13}C ($CDCl_3$) δ 211.38, 174.25, 160.20, 141.14, 130.01, 119.55, 113.38, 112.49, 69.70, 55.45, 51.65, 41.94, 39.92, 36.30, 34.04, 30.26, 27.79, 22.86, 22.56; MS *m*/*z* 334.30 (MH^+), Calculated for $C_{19}H_{27}NO_4$ (MH^+) 334.2013, found 334.2017.

Methyl 5-((2-oxo-1-(3-(trifluoromethyl)phenyl)cyclohexyl)amino)pentanoate (13b). Similar reaction of amine **30l** (0.26 g, 1.00 mmol) with methyl 5-bromovalerate (0.28 g, 1.42 mmol) gave **13b** (0.25 g, 67%), which was converted as above to the HCl salt. ^{1}HNMR ($CDCl_3$) δ 7.54–7.51 (m, 3H), 7.49–7.44 (m, 1H), 3.63 (s, 3H), 2.78–2.72 (m, 1H), 2.60–2.58 (t, *J* = 7.09 Hz, 1H), 2.52–2.46 (m, 1H), 2.40–2.36 (m, 1H), 2.30–2.22 (m, 4H), 2.04–1.99 (m, 2H), 1.98–1.82 (m, 6H), ^{13}C ($CDCl_3$) δ 210.63, 174.36, 141.23, 130.84, 129.43, 124.58, 124.47, 123.94, 69.64, 51.76, 41.98, 39.83, 37.18, 33.96, 30.18, 27.62, 22.74, 21.66; MS *m*/*z* 372.30 (MH^+), Calculated for $C_{19}H_{24}F_3NO_3$ (MH^+) 372.1793 found 372.1792.

Methyl 5-((2-oxo-1-(3-(trifluoromethoxy)phenyl)cyclohexyl)amino)pentanoate (14b). Similar reaction of amine **30o** (0.23 g, 0.84 mmol) with methyl 5-bromovalerate (0.33 g, 1.68 mmol) gave **14b** (0.26 g, 81%), which was converted as above to the HCl salt. ^{1}HNMR δ 7.42–7.36 (t, *J* = 8.12 Hz, 1H), 7.16–7.13 (m, 3H), 3.64 (s, 3H), 2.60–2.56 (t, *J* = 7.0 Hz, 1H), 2.50–2.44 (m, 1H), 2.40–2.22 (m, 4H), 2.00–1.70 (m, 6H), 1.63–1.54 (m, 2H), 1.44–1.32 (m, 2H); ^{13}C ($CDCl_3$) δ 210.60, 174.36, 149.93, 142.54, 130.30,125.56, 120.04, 119.99, 119.36, 69.64, 62.45, 51.76, 41.93, 39.80, 36.98, 34.09, 30.38, 27.62, 22.76, 22.30; MS *m*/*z* 388.20 (MH^+), Calculated for $C_{19}H_{25}F_3NO_4$ (MH^+) 388.1755 found 388.1753.

Methyl 5-((2-oxo-1-(*p*-tolyl)cyclohexyl)amino)pentanoate (16b). Similar reaction of amine **30g** (0.18 g, 0.88 mmol) with methyl 5-bromovalerate gave **16b** (0.20 g, 72%), which was converted as above to the solid HCl salt. ^{1}HNMR ($CDCl_3$) δ 7.18–7.15 (d, J = 8.04 Hz, 2H), 7.11–7.09 (d, *J* = 8.2 Hz, 2H), 3.62 (s, 3H), 2.88–2.82 (m, 1H), 2.41–2.28 (m, 2H), 2.34 (s, 3H), 2.23–2.20 (t, *J* = 7.4 Hz, 3H), 2.04–1.88 (m, 2H), 1.86–1.66 (m, 4H), 1.62–1.48 (m, 2 H), 1.46–1.32 (m, 2H); ^{13}C ($CDCl_3$) δ 211.79, 174.26 137.36, 136.49, 129.72, 127.12, 69.60, 51.64, 41.99, 39.88, 36.39, 34.07 30.31, 27.90, 22.89, 22.57, 21.26; MS *m*/*z* 318.20 (MH+), Calculated for $C_{19}H_{27}NO_3$ (MH^+) 318.2064, found 318.2065.

Methyl 5-((1-(4-methoxyphenyl)-2-oxocyclohexyl)amino)pentanoate (17b). Similar reaction of amine **30j** (0.10 g, 0.45 mmol) with methyl 5-bromovalerate (0.16 g, 0.59 mmol) gave **17b** (0.1 g, 66%), which was converted as above to the solid HCl salt. ^{1}HNMR ($CDCl_3$) δ 7.15–7.12 (d, *J* = 8.88 Hz, 2H), 6.91–6.88 (d, *J* = 8.88 Hz, 2H), 3.81 (s, 3H), 3.63 (s, 3H), 2.88–2.79 (m, 1H), 2.42–2.30 (m, 2H), 2.26–2.18 (t, *J* = 7.6 Hz, 3H), 2.06–2.00 (m, 1H), 1.98–1.90 (m, 1H), 1.86–1.78 (m, 2H), 1.78–1.68 (m, 2H), 1.56–1.48 (m, 2H), 1.48–1.32 (m, 2H); ^{13}C ($CDCl_3$) δ 211.83, 174.28, 158.98, 130.94, 128.46, 114.35, 69.36, 55.46, 51.66, 42.02, 39.82, 36.56, 34.06, 30.28, 27.91, 22.90, 22.58; MS *m*/*z* 334.20 (MH^+), Calculated for $C_{19}H_{27}NO_4$ (MH^+) 334.1945, found 334.2017.

Methyl 5-((2-oxo-1-(4-(trifluoromethyl)phenyl)cyclohexyl)amino)pentanoate (18b). Similar reaction of amine **30m** (0.18 g, 0.70 mmol) with methyl 5-bromovalerate (0.19 g, 0.98 mmol) gave **18b** (0.20 g, 76%), which was converted as above to the HCl salt. ^{1}HNMR ($CDCl_3$) δ 7.63–7.61 (d, *J* = 8.24 Hz, 2H), 7.38–7.36 (d, *J* = 8.16 Hz, 2H), 3.63 (s, 3H), 2.80–2.79 (m, 1H), 2.52–2.46 (m, 1H), 2.40–2.32 (m, 1H), 2.30–2.20 (m, 3H), 2.00–1.86 (m, 4H), 1.80–1.68 (m, 2H), 1.62–1.52 (m, 2H), 1.44–1.36 (m, 2H), ^{13}C ($CDCl_3$) δ 210.66, 174.17, 140.07, 127.70, 125.92, 69.66, 51.78, 41.99, 39.90, 36.98, 33.98, 30.22, 27.66, 22.76, 22.56; MS *m*/*z* 372.20 (MH^+), Calculated for $C_{19}H_{24}F_3NO_3$ (MH^+) 372.1781 found 372.1801.

Methyl 5-((2-oxo-1-(4-(trifluoromethoxy)phenyl)cyclohexyl)amino)pentanoate (19b). Similar reaction of amine **30p** (0.48 g, 1.76 mmol) with methyl 5-bromovalerate (0.48 g, 2.40 mmol) gave **19b** (0.54 g, 81%), which was converted as above to the HCl salt. ^{1}HNMR δ 7.28–7.26 (m, 2H), 7.21–7.19 (m, 2H), 3.63 (s, 3H), 2.78–2.72 (m, 1H), 2.49–2.42 (m, 1H), 2.37–2.22 (m, 4H), 2.03–1.94 (m, 3H), 1.88–1.72 (m, 3H), 1.61–1.53 (m, 2H), 1.49–1.33 (m, 2H), ^{13}C ($CDCl_3$) δ 211.00, 174.18, 148.55, 138.56, 129.05, 121.23, 69.31, 51.76, 41.98, 39.86, 37.02, 33.84, 30.24, 27.70, 22.79, 22.38; MS *m*/*z* 388.20 (MH^+), Calculated for $C_{19}H_{24}F_3NO_4$ (MH^+) 388.1755 found 388.1750.

Methyl 5-((2-oxo-1-(thiophen-2-yl)cyclohexyl)amino)pentanoate (20b). Similar reaction of nortiletamine (**35**) (0.17 g, 0.87 mmol) with methyl 5-bromovalerate (0.22 g, 1.1 mmol) gave **20b** (0.16 g, 62%), which was converted as above to the solid HCl salt. ^{1}HNMR ($CDCl_3$) δ 7.28–7.27 (dd, *J* = 5.1 Hz, 1.08 Hz, 1H), 6.99–6.97 (dd, *J* = 5.08 Hz, 3.56 Hz, 1H), 6.87–6.86 (dd, *J* = 3.56 Hz, 1.12 Hz, 1H), 3.64 (s, 3H), 2.68–2.64 (m, 1H), 2.52–2.46 (m, 2H), 2.38–2.30 (m, 1H), 2.28–2.24 (t, *J* = 3.04 Hz, 2H), 2.00–1.92 (m, 3H), 1.90–1.82 (m, 2H), 1.78–1.68 (m, 1H), 1.66–1.56 (q, *J* = 7.57 Hz, 2H), 1.52–1.38 (m, 2H); ^{13}C ($CDCl_3$) δ 209.76, 174.34, 145.62, 127.08, 125.89, 125.46, 67.68, 51.66, 42.20, 39.26, 39.11, 34.06, 30.20, 27.19, 22.89, 22.50; MS *m*/*z* 310.20 (MH^+), Calculated for $C_{16}H_{23}NO_3S$ (MH^+) 310.1471, found 310.1472.
